# A machine learning–based optimal charging strategy for PV-assisted electric vehicle systems incorporating second-life batteries under degradation constraints

**DOI:** 10.1038/s41598-026-60510-0

**Published:** 2026-07-07

**Authors:** Ahmed Ezzat, Ayman S. Abdel-Khalik, Ragi A. Hamdy, Mostafa S. Hamad

**Affiliations:** 1 Department of Electrical Engineering, Alexandria University, Alexandria, 21544 Egypt; 2 Department of Electrical and Control Engineering, Arab Academy for Science,Technology and Maritime Transport, Alexandria, 1029 Egypt; 3 Department of Electrical and Computer Engineering, , Sultan Qaboos University, 123 Muscat, Oman

**Keywords:** Energy management system (EMS), Second-life batteries, Battery degradation, Energy storage systems (ESS), Battery management systems (BMS), Energy science and technology, Engineering

## Abstract

The rapid expansion of electric vehicles (EVs) and residential photovoltaic (PV) systems has created new challenges in battery charging management, particularly due to the variability of renewable energy, grid limitations, and battery aging effects. In this work, we present a machine learning–based Energy Management System (EMS) designed for PV-assisted smart charging of EVs and second-life batteries. The proposed system estimates the Optimal Charging Duration Class (OCDC) using an XGBoost model trained on real-time operating variables such as state of charge (SOC), battery temperature, available PV surplus, and degradation-related indicators. Rather than relying on conventional continuous power control, the proposed approach adopts discrete charging modes that dynamically adjust to operating conditions, aiming to improve both energy utilization and battery health. Degradation considerations are incorporated in a practical, control-oriented manner by avoiding operating regions associated with accelerated aging, instead of explicitly modeling electrochemical processes. The system is evaluated within a simulation framework that includes realistic PV generation profiles, load demand, and thermal behavior. Although hardware implementation is not yet included, it is identified as an important direction for future validation. The results show that the proposed EMS increases PV self-consumption by around 22% while reducing exposure to high-temperature operation, high state-of-charge conditions, and unnecessary cycling. These outcomes indicate a potential reduction in degradation risk, especially for second-life battery applications. Overall, the study demonstrates that integrating machine learning with real-time energy management can provide an efficient and health-aware solution for smart charging in residential and hybrid renewable energy systems.

## Introduction

The rise of Electric Vehicles (EVs) is an essential part of the ongoing transformation of the transport sector and the global effort to reduce greenhouse gas emission levels. The transition to Electric Vehicles is also integrally tied to the transition to sustainable energy systems, which requires an increased percentage of variable renewable energy sources being incorporated into the electrical grid. The vision for this is an electric mobility system that produces no direct emissions, not just the transfer of emissions created from vehicle operation to the generation of electric power. However, the rapid and concurrent rise in both Battery Electric Vehicle (BEV) adoption and variable renewable energy (VRE) injection has introduced significant challenges from both a technical and an economic perspective^1^.

Existing EV charging strategies provide minimal opportunity to optimize multiple objectives simultaneously (charging time, battery health, and the variability associated with photovoltaic (PV) generation) because they are primarily reliant on static scheduling and cost-based optimization methods that do not take into account long-term degradation of the battery and do not utilize the real-time opportunity associated with the instantaneous availability^2^. The increasing adoption of EVs also introduces complex challenges related to battery aging and capacity degradation, which directly affect driving range and residual vehicle value. As millions of EVs are charged simultaneously, significant stress is placed on power grids, potentially leading to voltage fluctuations, localized outages, and transformer overloading^3^.

Smart charging provides an excellent solution to help utilities gain real-time communication with electric vehicles (EVs). Smart charging utilizes advanced analytics to create optimal charging schedules, taking into account grid conditions (i.e., the state of the electric system in terms of demand and supply), pricing for electric energy, and access to renewable sources of energy, and aligning the demand for EV charging with times when surplus solar and/or wind energy is available. Smart charging enables the sustainability of electric mobility by reducing the associated carbon emissions of EVs^4^. An example of this type of sustainability initiative is the second-life battery (SLB) applications of reusing EV battery systems after they have degraded (i.e., are operating at approximately 70–80% of their original capacity). Although EV batteries can no longer meet automotive performance standards, they provide a viable source of energy storage as they can be reused in stationary energy storage systems, to support the integration of renewable energy into the grid, to support grid support services, and as backup energy systems. By re-using EV batteries whenever possible, the amount of waste produced will be reduced, and the lifecycle cost will be lowered, thus providing further sustainability of energy storage. Nevertheless, challenges in determining the health of used EV batteries, developing standard procedures for assessing their health, and ensuring safe large-scale deployment exist^5,6^.

As variable renewable energies continue to grow and the greater use of electric vehicles (EVs) spreads, there will be an increased need for accurately controlled charging times so that grid stability is maintained, costs are minimized, and battery health is preserved. By implementing optimized charging schedules, the potential exists to reduce peak demand as energy use is transferred from times of high cost or carbon output and to install EV charging during times when renewable energy is readily available, thus reducing both operational stress on the grid and emissions. While progress has been made in a controlled laboratory environment, the lack of validated real-time battery degradation models that can be used by commercial smart charging systems continues to hinder progress in applying this capability in the real world^7^.

Most of the existing approaches that have been used to develop EV charging stations are based on simplified assumptions, using models that do not adequately consider the interaction of driving behavior, charging conditions, environmental conditions and battery chemistry. Further, there are no existing systems that provide battery health data in a format that can be implemented into common grid communication protocols. As such, most current smart charging systems are incapable of providing cost-effective, grid-friendly, and battery-friendly solutions^8^. This paper presents a deployable ML-integrated EMS architecture that incorporates an optimized charging duration prediction model into real-time PV-aware control for both EV and second-life battery applications. The main contributions of this work are as follows:

- A deployable ML-integrated EMS architecture for PV-aware smart charging of EV and second-life batteries, enabling real-time, health-aware closed-loop control.
- An XGBoost-based Optimal Charging Duration Class (OCDC) prediction module embedded within the EMS decision framework to dynamically adjust charging behavior based on state of charge (SOC), temperature, PV energy surplus, and battery degradation indicators.
- A robust evaluation framework combining k-fold cross-validation, confidence intervals, and SHAP-based interpretability analysis to ensure reliable, leakage-free model deployment.
- A system-level validation demonstrating improvements in PV energy utilization, reduction in battery thermal stress, and enhanced lifecycle cost efficiency, particularly for second-life battery applications.

The subsequent sections of the paper are structured as follows: In  “Literature review and related work” section: Current research on using machine learning for application to battery systems; In “The proposed framework” section: The proposed framework and methodology; In “Experimental work” section: Experimentation setup, including the preprocessing of the data sets and the analysis of ML models; In “Applied XGBoost for PV-smart ESS charging” section: The implementation of the XGBoost model in a photovoltaic integrated smart energy storage system; and “Conclusion” section: Conclusions and future work with regards to the results of this research.

## Literature review and related work

The growing adoption of EVs and renewable energy resources has created new opportunities for energy management while simultaneously introducing complex challenges. Smart charging represents a promising pathway toward more sustainable and cost-effective electric mobility; however, battery degradation remains a critical concern, as suboptimal charging strategies may accelerate aging and reduce battery service life. Conventional approaches typically rely on fixed schedules or static optimization frameworks that fail to account for dynamic operating conditions or the long-term impact of charging patterns on battery health^4^. Furthermore, conventional approaches lack the predictive capabilities required to respond dynamically to variable renewable energy availability when determining charging duration. The development of an intelligent, data-driven framework is therefore necessary to balance battery longevity, energy efficiency, and renewable energy integration^9^.

Duraisamy et al.^10^ propose a machine-learning-driven passive cell-balancing approach that adaptively selects shunt resistor values to minimize balancing time and thermal stress, outperforming conventional fixed-resistor designs. Chacko et al. created an Intelligent Energy Management System (IEMS) for plug-in hybrid electric vehicles (PHEVs). This system allows for the sharing of energy between vehicles and the grid (vehicle-to-grid (V2G) and between vehicles themselves (vehicle-to-vehicle (V2V), using a multi-objective predictive optimization approach. With this system, they were able to support microgrids without affecting the vehicle’s ability to drive^11^.

Shen et al.^12^ developed a power estimator based on nonlinear optimization with sensitivity analysis. This method provides accurate power limits regardless of the variability of conditions by determining the critical parameters. They presented a shift from using static, rule-based approaches to adaptive, data-driven approaches that will both maximize energy efficiency and extend battery life and also allow for the integration of renewable energy. The challenge now is to scale up the systems, reduce their computational complexity, and validate them in real-world applications across multiple architectures.

Rivera et al.^13^ performed a comparison of three energy storage technologies (Li-ion batteries, Vanadium Redox Flow Batteries “VRFB’s”, and Hydrogen Storage) as methods to capture spillable turbine-able electricity from a large hydroelectric facility. Simulation results indicate all three methods can provide the required time presence for an energy shift; however, each performs better than others under different criteria. The scientific value associated with this research is due to its comprehensive approach with multiple alternative criteria in combination with opportunities for Li-ion (specifically second-life EV batteries) to compete for the ability to take advantage of arbitrage in large-scale electricity trading markets, therefore providing support for Li-ion as an integral component of circular energy systems. The overall study is descriptive at the macro level from a techno-economic standpoint and does not provide details regarding the various technical issues related to BMSs, including estimating SOC and SoH, and balancing cells, which limits the direct applicability of their work with respect to BMS-related studies.

Guo & Shen^14^ introduce a data-model fusion framework for online state-of-power (SoP) estimation that combines physics-based models with data-driven corrections to improve accuracy under high discharge rates during transient power use; however, this approach requires extensive training data at high rates to validate the data. Cabrera-Castillo et al. (2016) provide a state-of-safety (SOS) metric that integrates voltage/current/temperature/pressure into a single SOS metric, providing the foundation for a proactive BMS specific to safety protection strategies; however, no implementation exists for real-time SOS calculation^15^. Zhang & Zhang^16^ have developed a joint MAE and state-of-energy (SoE) estimation method across a temperature range; although this improved power management/estimation indicator methods for energy but still requires extensive thermal parameter mapping as part of the new method.

Petri & Petreu^17^ developed an EV BMS prototype that included basic hardware for energy balancing with SOC estimation. The prototype tests have been validated through experimentation to ensure the cells maintain consistent voltages during operation and for acceptable SOC accuracy. The SOC estimation has no SoH modelling or advanced thermal considerations as part of the design. Breglio et al.^18^ further developed the prototype by adding active balancing and developing a SOC/SoH modelling system using degradation-aware observers to redistribute energy more intelligently. The coupling of active and model-based SOC/SoH estimation processes would result in increased performance and longevity but would require a significant amount of computational resources. Uzair et al.^19^ provided a review of the various BMS architectures and balancing topology options, developed a taxonomy of both active and passive methods, and compared each methodology according to its operational efficiency and design complexity, enabling comparison against each method.

Kosuru & Venkitaraman^20^ presented a smart BMS sensor that utilizes deep learning for sensor fault detection to enable BMS systems to operate effectively, despite corrupted sensor measurements. Although smart BMS is reliable, challenges exist for large-scale deployment. Ragone et al.^21^ developed an energy-based SOC estimation process using a synthetic dataset generated from multi-physics battery modelling and automotive simulations; their performance was good, but they did not include SoH or energy balancing in their estimation approach.

Yang et al.^22^ build on a CHAIN-based cloud BMS architecture, which is designed to provide reliable data for remote monitoring and usage, but does not offer much sophistication in terms of the algorithms used in the cloud component of the BMS. Bhovi et al.^23^ provided a simulated EV BMS including SOC estimation and protection logic for the preliminary design of a BMS; however, there was no experimental data to validate the simulation, and there was no advanced equalization in the simulation. Sarker et al.^24^ modelled the integration of second-life batteries into residential PV systems, finding benefits from an economic and environmental perspective, but only using simplified assumptions on the BMS used to facilitate the connection of the second-life battery to the PV system.

The balancing techniques discussed in the above papers include passive balancing driven by machine learning (based on the work of Duraisamy), as well as integrated SOC balancing prototypes (Petri & Petreu) and model-based active balancing with a SoH-aware control (Breglio). An overview of both active and passive methods is provided by the work of Uzair. Examples of advancement in state estimation include SOC (Takyi-, Ragone), SoH through balancing currents (Tang), SoP for high discharge using a hybrid data-model fusion technique (Guo & Shen), and energy-centric indices such as Remaining Available Energy (Chen) and Maximum Available Energy/SoE (Zhang & Zhang), along with safety-focused metrics such as State of Safety (Cabrera-Castillo). Works on energy management and smart charging (Chacko, Rivera) include predictive optimizations and second-life integration for renewable systems. Intelligent and cloud-enabled BMS architectures (Shi, Yang, Bhovi, Kosuru) feature AI-based fault detection, digital twins, and fleet-level optimization, while second-life applications for the circular economy. Sarker investigated the potential to reuse storage and solar PV systems. However, a common limitation across these works is the absence of real-time ML-based charging control tailored specifically for second-life battery systems integrated with residential PV. Sarker et al.^24^ address second-life batteries in PV systems but rely on simplified BMS assumptions without real-time adaptive control. Rivera et al.^13^ evaluate second-life storage from a techno-economic standpoint without addressing degradation-aware BMS-level decision making. To the best of the authors’ knowledge, the integration of an interpretable ML classifier into a closed-loop EMS specifically designed for health-aware charging of second-life batteries in PV-assisted systems has not been previously demonstrated in the literature, which motivates the present work.

## The proposed framework

**Fig. 1:**
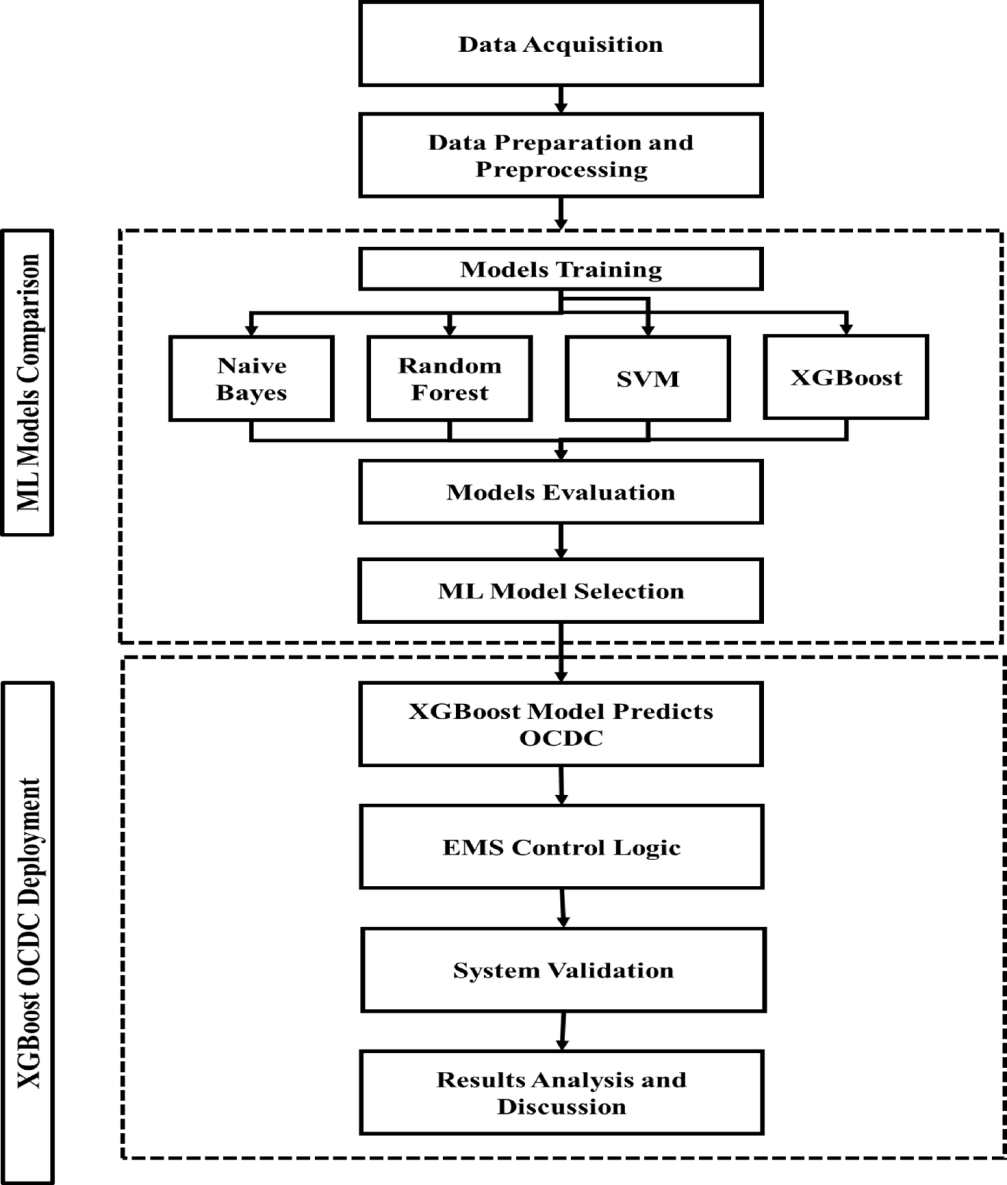
The proposed framework of the research methodology.

The research proceeded in two phases, as shown in Fig. 1, phase 1 benchmark Naive Bayes, Random Forest, XGBoost, and SVM via accuracy, precision/recall/F1, confusion matrices, and t-tests on an Excel dataset of operational variables (SOC, temperature, PV surplus, and charging-duration class). XGBoost was the best classifier because of its high accuracy, low “Short” class misclassification, robust modeling of nonlinear relationships (SOC–temperature–PV excess), and quick inference for real-time BMS/EMS. In Phase 2, the XGBoost Optimal Charging Duration Class (OCDC) model was integrated into a PV-Smart Energy Management System (EMS) for second-life EV batteries, including PV generation, load model, Energy Storage System (ESS) pack, OCDC decision engine, and charge-power modulation updated every 15–30 min. The EMS used real-time signals (PV power, load, SOC, temperature, and SoH) to map OCDC outputs to charge multipliers (S: 0.2, M: 0.5, L: 1.0) in a 24-hour, clear-sky scenario (5 kW PV, 20 kWh ESS, initial SOC 40%, variable load). The results showed realistic class transitions (S→M→L in the morning, M at midday for thermal moderation, S near SOC ≈ 95%, disabled when PV < 0). XGBoost-driven OCDCs showed 22% higher PV utilization, reduced depth-of-discharge and thermal stress, prevented high-SOC overcharge, and improved SOC trajectory with healthier C-rates, proving their suitability for second-life ESS deployments.

The ML algorithms, especially XGBoost, RF over ANN, and LSTM deep learning models, were largely based on the inherent suitability of the XGBoost algorithm for structured tabular data such as battery data. In addition to being more accurate/stable than deep learning algorithms, XGBoost does not require large amounts of training data or complex extraction processes for features from the data. The very low computational cost and sub-millisecond inferencing latency of XGBoost models allow for real-time operation on limited-resource embedded hardware that supports EMS and BMS. In contrast, deep learning models impose excessive memory and processing requirements on this type of hardware. XGBoost provides transparent interpretability via SHAP analysis (Shapley additive explanations) to ensure that charging decisions will remain physically interpretable and reasonable with respect to the health of batteries; this is critical for safety-critical energy storage systems. In addition to providing high classification performance and being resistant to overfitting, XGBoost thus provides a practical, efficient, and explainable model to meet the primary objectives of this research project better than any alternative deep learning models.

## Experimental work

The experiments for this machine learning research were conducted using an EV battery charging database that is available to the public and can be downloaded from Kaggle. This database contains a large number of charging telemetry records with their associated variable data (both electrical and thermal data) that describe the various charging sessions of EVs and how each of the variables impacts the aging of batteries and how the batteries are charged. The attributes of the data set make it extremely well-suited for deriving and testing OCDC predictive models.

### Dataset structure

The dataset used in this study is obtained from a publicly available Kaggle repository^1^. It contains EV battery charging session records with time-resolved electrical, thermal, and operational features. Some of the primary state variables that will be measured in this study will be SOC (%), volts (V), and amps (A), while allowing the ability to reflect the energy present in the battery, the internal resistance of the battery, and the charging rate of the battery. The thermal measurements of battery temperature and ambient temperature will also provide a basis for determining degradation risk and thermal behavior. The metadata associated with the charging records will provide context for the associated charging sessions by reporting information such as charging power (kW) or efficiency (%), type of batteries, number of cycles the battery has been charged, and the vehicle model that was charged.

The target variable, Optimal Charging Decision Class (OCDC), is not explicitly contained within the original Kaggle dataset; rather, it is synthesized via a rule-based labeling framework utilizing domain-specific heuristic thresholds to emulate authentic battery management strategies. The OCDC labels were generated using an ordered rule-based procedure. Class S (Short/protective mode) is assigned when any one of the following protective conditions is met: SOC exceeds 90%, battery temperature exceeds 40 °C, or PV surplus is below 0.5 kW. Among the remaining non-protective samples, Class L (Long/aggressive mode) is assigned when SOC is below 80%, battery temperature is within the safe range, and PV surplus exceeds 2.0 kW. All other samples are assigned to Class M (Medium/balanced mode). This ordered procedure makes the label-generation process deterministic and exhaustive. By systematically mapping these operational states into discrete classes, this strategy establishes a robust ground truth suitable for supervised learning tasks.

The EV battery charging dataset maintains an adequate level of integrity, as it has a low number of missing entries among continuous numerical type variables. However, it requires normalization due to the wide range of values for SOC, voltages, currents, and temperatures, and their non-uniform scaling. Minor discrepancies in the means of several features (associated with SOC, voltage, current, and temperature) were resolved through standardization, label encoding, and splitting the dataset into a train/validation/test set. Outliers were verified based on the current-to-temperature ratio.

The dataset will provide an adequate basis for using predictive modelling with sophisticated algorithms such as Naive Bayes, Random Forests, SVMs, and XGBoost, enabling adequate classification of various charging duration classes. Furthermore, the attributes of the dataset are like those attributes measured and monitored for use in BMS for use in EV operating in conjunction with an Energy Storage System (ESS) PV-integrated battery second life. Therefore, the trained XGBoost model can be seamlessly integrated into existing Energy Management Systems, which foster sustainability. This dataset contains features that will contribute directly towards predicting optimal charging behavior and battery health based upon SOC, temperature, voltage, and current, as they all have a significant impact on a battery’s long-term performance and degradation risk.

High SOC greater than 90%, high temperature (greater than 40 °C), or low PV surplus (below 0.5 kW) — any one of these conditions alone is sufficient to trigger protective Class S status, allowing the battery to prioritize protection and charge at minimal intensity. The OCDC Class M status is where the battery is operating under normal PV surplus (greater than 80%) with a mid-range SOC of 80–90%. This system provides balanced charging of the battery. Finally, OCDC Class L has a high PV surplus (less than 80%) with low to mid SOC (less than 80%) and safe operating temperatures; therefore, the system is able to take advantage of the most renewable energy available for charging the battery.

**Fig. 2:**
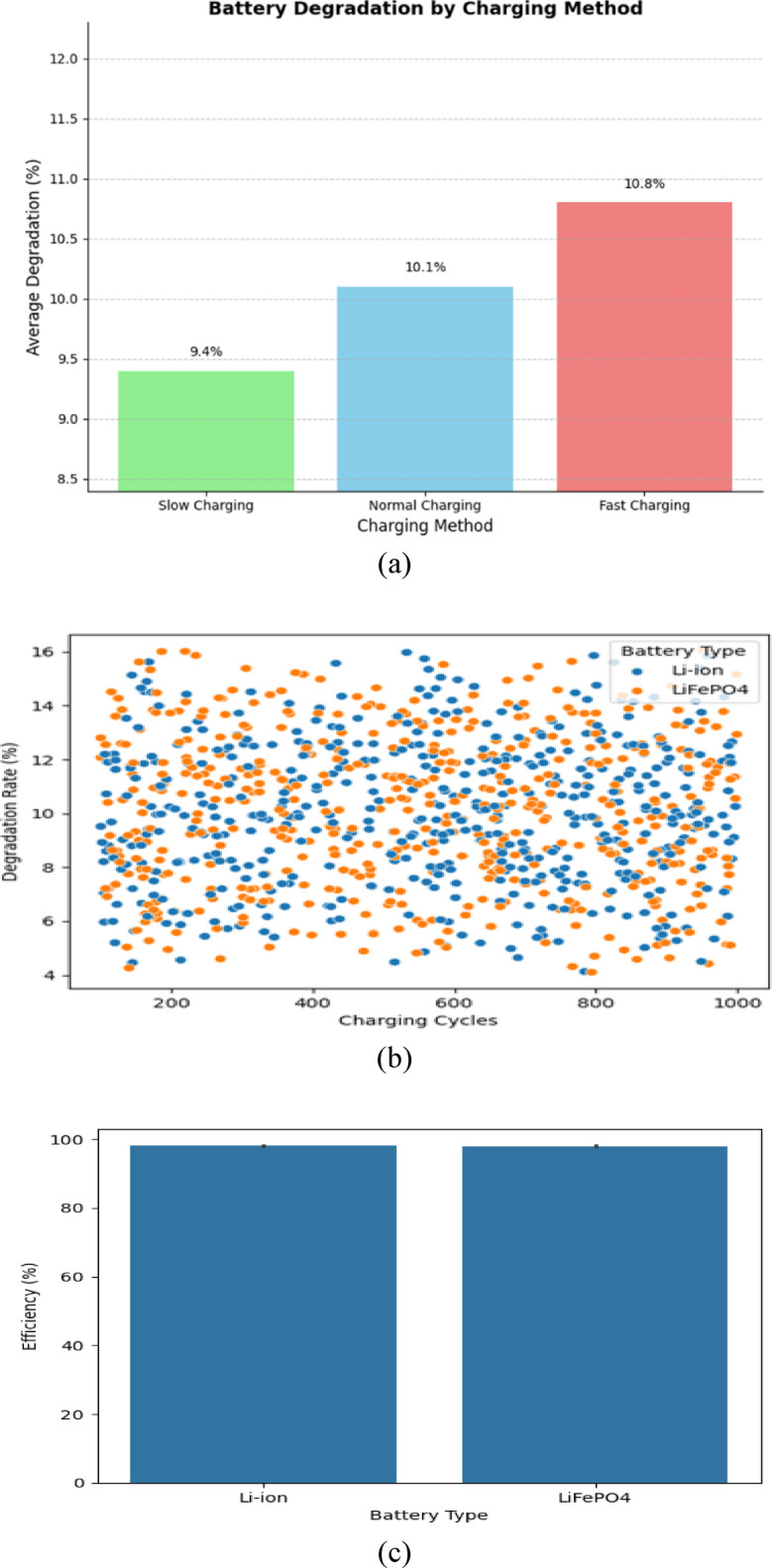
(**a**) Average battery degradation under different charging methods, (**b**) Scatter plot of degradation rate versus charging cycles for Li-ion and LiFePO₄ batteries (left), and (**c**) comparison of charging efficiency between the two chemistries (right).

The average degradation rates are associated with three common charging strategies: slow charging, normal charging, and fast charging, as shown in Fig. 2a. The results highlight a clear trend where faster charging correlates with higher degradation levels. The relationship between degradation rate and charging cycles for two battery chemistries: Li-ion and LiFePO₄. Although both chemistries exhibit a wide dispersion across the dataset, Li-ion cells tend to show slightly higher degradation variance, consistent with their greater sensitivity to thermal and C-rate conditions, as shown in Fig. 2b. The bar chart on the right compares the charging efficiency of both chemistries, showing nearly identical efficiency values (~ 98–99%), as shown in Fig. 2c.

**Fig. 3:**
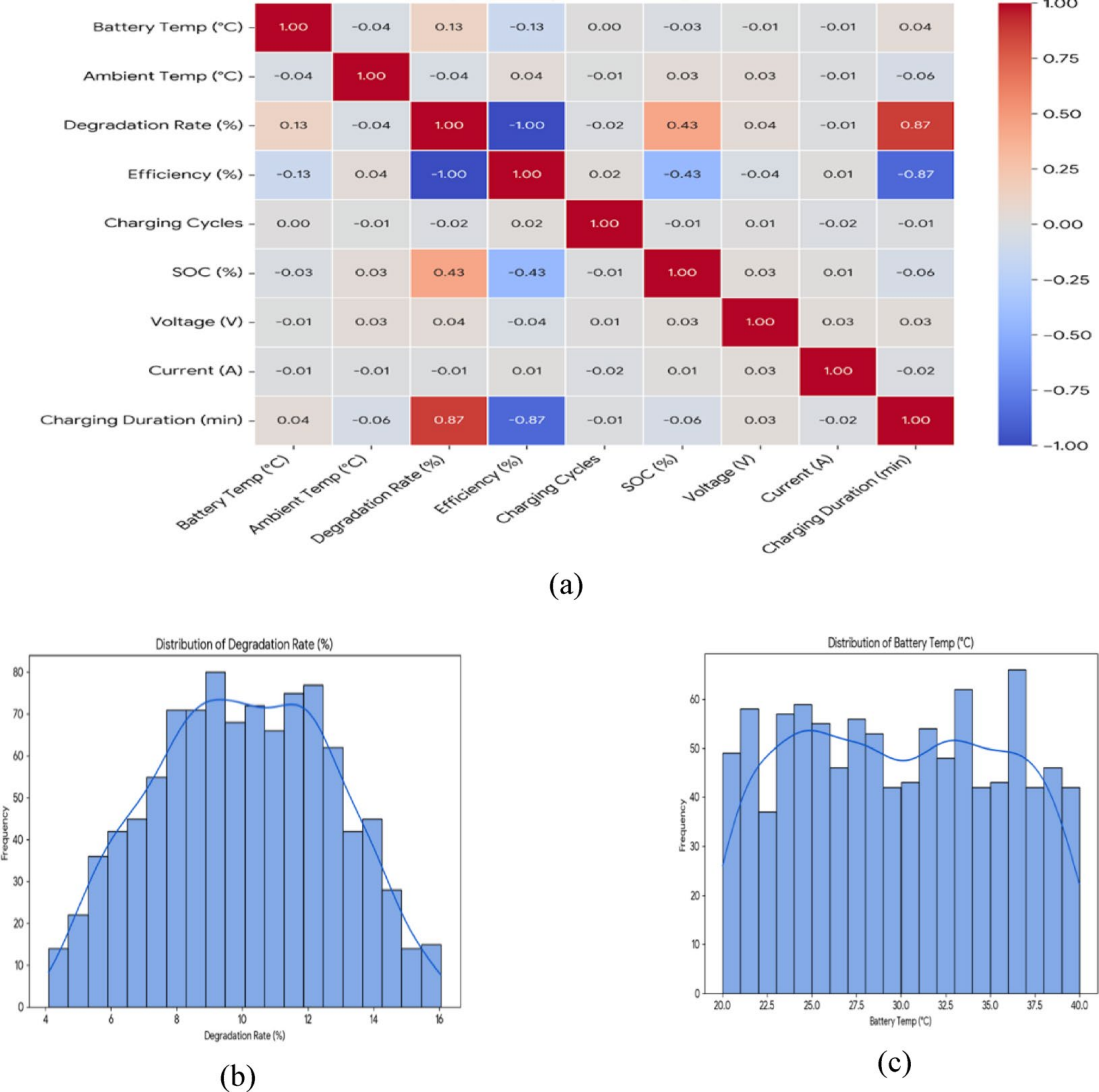
(**a**) Correlation matrix showing relationships among dataset parameters, (**b**) Histogram and density plot showing the distribution of degradation rates across all samples, and (**c**) Distribution of battery temperature values.

The correlation heatmap reveals several key interactions between thermal, electrical, and degradation, as shown in Fig. 3a. Battery temperature shows a moderate positive correlation with degradation rate. Charging duration exhibits a strong correlation with both degradation rate and efficiency. Battery State of Charge and its moderate correlation to battery degradation indicate that longer discharge cycles may contribute to a battery deteriorating sooner than it would if it had only been discharged for shorter periods. Also, the heatmap provides important information about the interdependence of the variables, which is useful when selecting variables for machine learning model development. The degradation rate distribution is close to normal (approximately Gaussian shape) and follows this shape reasonably closely. As indicated in Fig. 3b, most values fall between 8% and 12%. The density curve also suggests that the degradation rate is centered at (mean) approximately 10%, which is consistent with what would be expected from the performance of a battery at the mid-life of an EV. The data indicate that the temperature of the battery mostly occurs between 22° and 36 °C and is uniformly distributed across the data, as represented in Fig. 3c.

### Dataset preparation and feature engineering

The first step in the data preparation process is to identify missing values, remove outliers from the data, and ensure there are no inconsistencies or unrealistic values within the data. All numerical variables, including state of charge, battery temperature, ambient temperature, voltage, current, and degradation rate, have been normalized on a common scale to prevent bias in the model due to the magnitude of the inputs. All categorical attributes (battery type, charging method, EV model, etc.) were also transformed using a label encoder for compatibility with tree-based and kernel-based algorithms. The target variable OCDC has been encoded into three categories: short, medium, and long. PV surplus (PV generation - household load) was created as a new feature in the feature engineering phase, and variables related to temperature were aggregated to provide additional predictive relevance. A correlation analysis and distribution profile were used to determine which of the independent variables would be the most informative for training the models.

To prevent target leakage and ensure robust model generalization, the primary features utilized to define the Optimal Charging Decision Class (OCDC), namely state of charge (SOC), photovoltaic (PV) surplus, and temperature thresholds, were strictly controlled during training. Rather than explicitly encoding the rule-based boundaries or incorporating derived binary flags that would lead to trivial rule memorization, the models were trained exclusively on the continuous, raw variables. This strategic exclusion forces the system to learn nuanced, generalizable operational patterns rather than deterministic labeling heuristics, thereby preserving the integrity of the evaluation metrics. The EV Battery Dataset was divided into training and testing datasets in an 80/20 ratio, ensuring that the different classes in the dataset were represented equally. This allowed for the accurate evaluation of Naive Bayes, Random Forest, SVM, and XGBoost.

### Training and evaluation of machine learning models

**Fig. 4:**
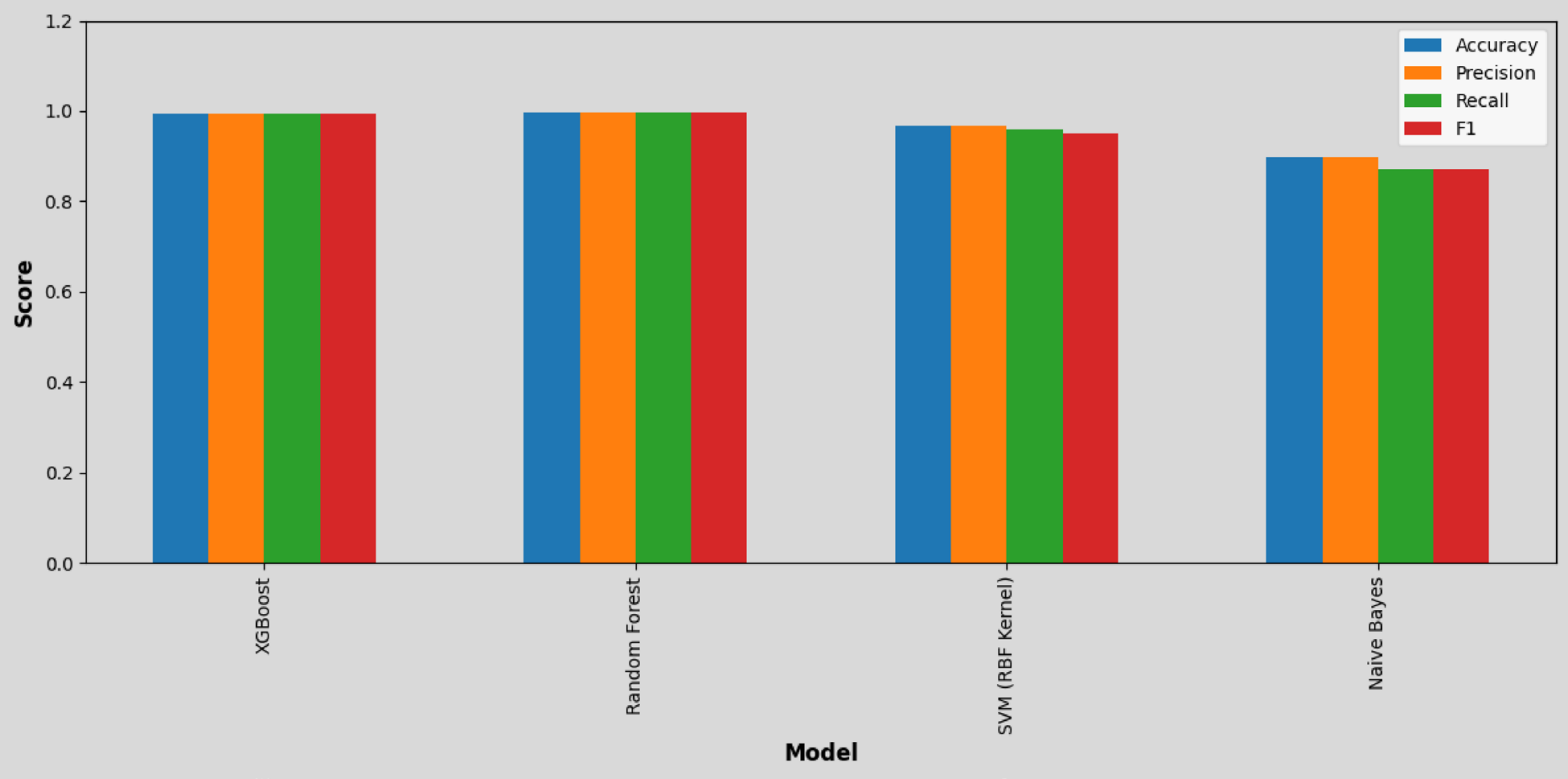
Comparison of model performance based on Accuracy and F1-score for XGBoost, Random Forest, SVM (RBF kernel), and Naive Bayes. XGBoost and Random Forest.

The four supervised machine learning models—Naive Bayes, Random Forest, Support Vector Machine (SVM), and XGBoost were developed to classify the Optimal Charging Duration Class (OCDC) using photovoltaic-assisted battery operational data. The dataset was preprocessed and then split into training and testing subsets using an 80/20 ratio, while maintaining class distribution across the three defined categories (Short, Medium, and Long). During model training, nonlinear dependencies between PV surplus, state of charge (SOC), battery temperature, electrical variables, and degradation-related indicators were learned by the models. Hyperparameter tuning was performed for Random Forest (number of trees and depth), SVM (kernel type and regularization), and XGBoost (learning rate, tree depth, and boosting iterations) using grid search combined with cross-validation. Naive Bayes was used as a reference baseline due to its simplicity. Model performance was evaluated using standard classification metrics, including accuracy, precision, recall, F1-score, and confusion matrices. As illustrated in Fig. 4, both XGBoost and Random Forest delivered the best performance, with cross-validation accuracies of approximately 0.995 and 0.999, respectively. SVM achieved slightly lower performance (Accuracy ≈ 0.967, F1 ≈ 0.968), while Naive Bayes showed the weakest results (≈ 0.87), which is expected given its assumption of conditional feature independence. The superior performance of the ensemble methods can be explained by the nature of the OCDC formulation, where decision boundaries are largely driven by threshold-based rules and nonlinear interactions among physically meaningful variables. This structure aligns well with tree-based learning approaches. To ensure a reliable evaluation, the dataset split was performed prior to training, and stratified cross-validation was applied. In addition, care was taken to avoid any overlap between training and testing samples, and features that could directly encode the labeling logic were excluded. Overall, the classifier is intended as a lightweight decision-support tool within the EMS, enabling fast approximation of optimal charging decisions without introducing significant computational overhead.

**Fig. 5:**
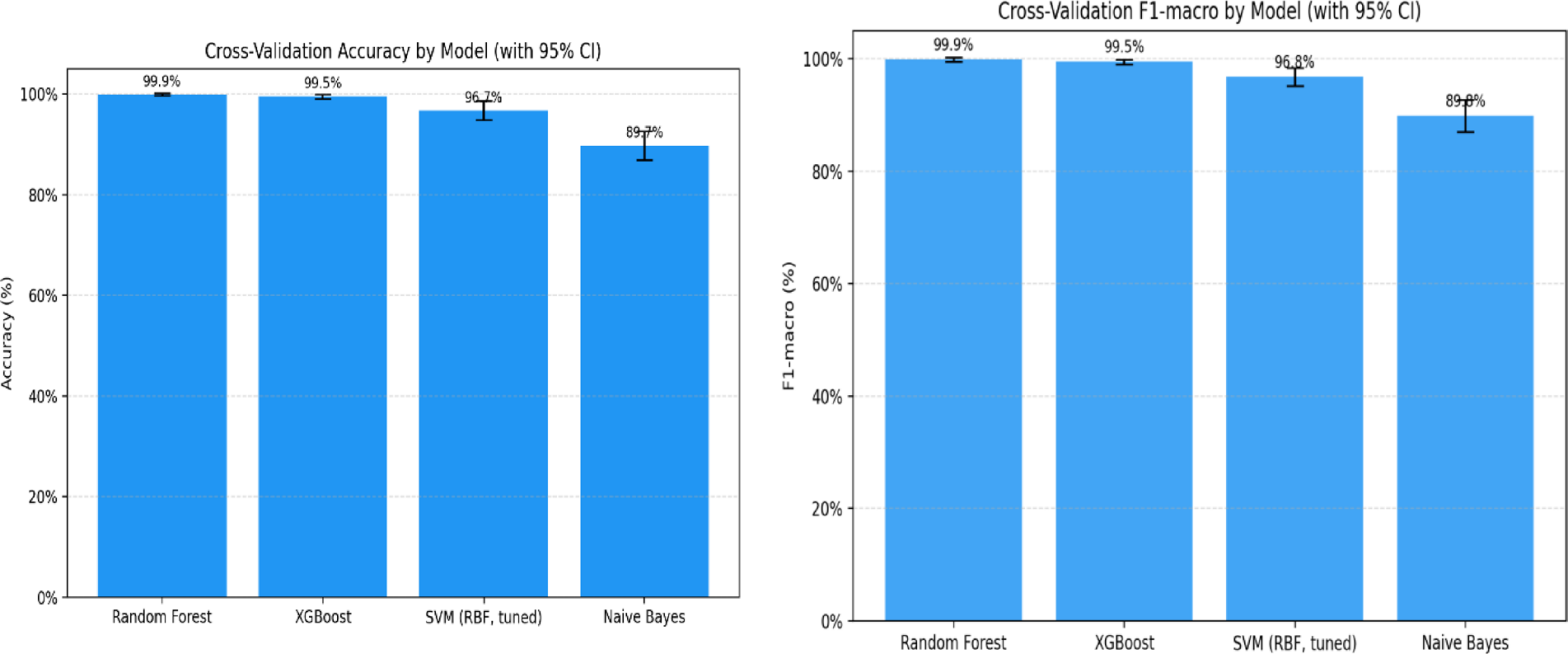
Comparative cross‑validation performance of machine‑learning models for optimal charging duration prediction. (**a**) Mean classification accuracy and (**b**) mean F1‑macro score.

**Fig. 6:**
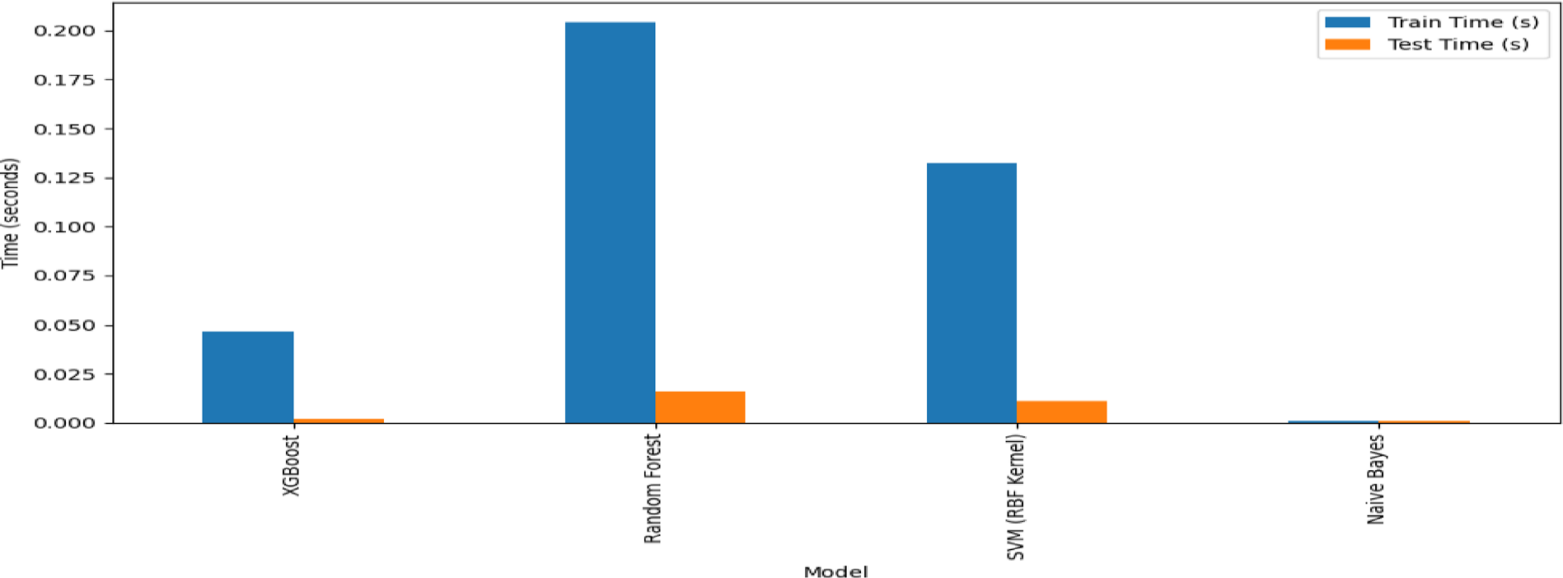
Comparison of training and testing times for the ML models.

As shown in Figs. 4 and 5, both XGBoost and Random Forest consistently achieve very high accuracy, precision, recall, and F1-scores. This behavior reflects the structure of the OCDC formulation, where class boundaries are largely defined by deterministic thresholds on state of charge (SOC), temperature, and PV surplus. Since the OCDC labels are generated using rule-based conditions, the mapping between input features and output classes is relatively well structured. This makes the problem particularly suitable for tree-based ensemble methods, which are designed to capture nonlinear and threshold-driven relationships. As a result, Random Forest and XGBoost outperform Naive Bayes and SVM across all metrics. A SHAP-based analysis was also conducted to improve interpretability. The results confirm that feature contributions remain consistent across different folds, indicating stable model behavior. In addition, the low variance observed in cross-validation results (Fig. 5) suggests that the performance is stable across data splits and not dependent on a particular partition. The computational analysis in Fig. 6 shows clear differences in efficiency among the models. Naive Bayes has the lowest computational cost, while XGBoost maintains a good balance between performance and efficiency, with very low inference time (< 0.005 s) and relatively short training time (~ 0.045 s). Random Forest, despite its high accuracy, requires significantly more training and inference time due to its large number of trees, which may limit its use in real-time EMS applications. SVM shows moderate training complexity and slower inference compared to XGBoost. Based on the combined results, XGBoost was selected for deployment due to its balance between accuracy and computational efficiency, making it more suitable for real-time EMS implementation. Its gradient boosting structure is also well aligned with the nonlinear and threshold-based nature of battery charging decisions. The high overall performance is partly influenced by the structured nature of the dataset and the clear separability of operating conditions (Table 1).

**Table 1:** The Cross-validated performance between evaluated methods.

Model	CV Accuracy	CV F1-score
Random Forest	0.999	0.998
XGBoost	0.995	0.994
SVM (RBF, tuned)	0.967	0.968
Naive Bayes	0.897	0.897

A comparative evaluation of four supervised learning algorithms—Naive Bayes (NB), Random Forest (RF), Support Vector Machine (SVM), and eXtreme Gradient Boosting (XGBoost) was conducted to identify the most suitable model for predicting the Optimal Charging Duration Class (OCDC). The assessment considered cross-validation accuracy, holdout set performance (accuracy, precision, recall, and F1-score), and computational cost. Both XGBoost and Random Forest achieved consistently high performance across all evaluation metrics, outperforming Naive Bayes and SVM by a clear margin. However, XGBoost demonstrated slightly better overall behavior in terms of training stability across folds, as well as lower inference time and more efficient training compared to Random Forest. In contrast, Naive Bayes and SVM showed lower predictive performance, particularly in distinguishing between medium and long charging duration classes. This limitation suggests that these models are less capable of capturing the nonlinear and interdependent relationships present in PV–battery operational data. Based on this trade-off between accuracy, stability, and computational efficiency, XGBoost was selected as the final model for integration within the EMS framework, particularly due to its suitability for real-time and embedded applications where latency and generalization are critical.

#### Noise-based robustness validation

To further examine the robustness of the proposed classification framework under practical measurement uncertainty, a sensor-noise sensitivity analysis was considered. In real EMS and BMS operation, input variables such as battery state of charge, temperature, PV power, current, and voltage are subject to measurement errors, estimation uncertainty, and signal fluctuations. Therefore, representative noise levels were defined for the main real-time input variables, as summarized in Table 2. These perturbations are intended to emulate realistic sensor uncertainty and assess whether the trained models remain stable when the input feature space is slightly disturbed. Three levels of noise were considered: low, moderate, and high noise. The low- and moderate-noise cases represent typical measurement uncertainty, whereas the high-noise case represents a stress-test condition near operational decision boundaries.

To evaluate the robustness of the proposed EMS under practical measurement uncertainty, Gaussian noise was injected into the main real-time input variables used by the classifier. Gaussian noise was selected because it provides a simple and commonly used representation of random sensor measurement errors and estimation uncertainty. The noisy input signal was defined as:

$$\:{x}_{\mathrm{noise\:}}\left(t\right)=x\left(t\right)+\varepsilon$$

where: $\:\varepsilon\sim\:\mathcal{N}\left(0,{\sigma\:}^{2}\right)$, x(t) represents the original measured input variable at time t, $\:{x}_{\mathrm{noise}}\left(t\right)$ represents the perturbed noisy input, and ε is a zero-mean Gaussian random variable with variance $\:{\sigma\:}^{2}$ Different values of σ\sigmaσ were selected according to the assumed uncertainty level of each sensor signal. Three noise scenarios were considered: low noise, moderate noise, and high noise. The low-noise case represents normal sensor uncertainty, the moderate-noise case represents realistic operating uncertainty, and the high-noise case represents a stress-test condition.

**Table 2:** Sensor-noise levels considered for robustness analysis of the EMS input variables.

Scenario	SOC noise	Temperature noise	PV surplus noise	Description
N1 – Low noise	± 1%	± 0.5 °C	± 2%	Normal sensor uncertainty
N2 – Moderate noise	± 2%	± 1 °C	± 5%	Realistic operating uncertainty
N3 – High noise	± 5%	± 2 °C	± 10%	Stress-test condition

To reduce the influence of random perturbation patterns and obtain statistically stable robustness estimates, each noise scenario was repeated 30 times using Monte Carlo trials. In each repetition, independent random noise was injected into the selected input variables according to the predefined noise levels. The classification performance was then re-evaluated, and the mean and standard deviation of accuracy and macro F1-score were reported across the 30 runs. For the EMS-level evaluation, the corresponding mean and standard deviation of key operational metrics, including PV utilization, peak grid draw, Thermal Stress Index, and final SOC, were also computed.

To further assess whether the high classification accuracy is robust under non-ideal measurement conditions, the trained models were re-evaluated after injecting different levels of sensor noise into the main input variables. As shown in Table 3, all models experienced a gradual reduction in accuracy as the noise level increased, which is expected because noisy measurements can move samples closer to or across OCDC decision boundaries. However, Random Forest and XGBoost maintained high accuracy even under high-noise conditions, achieving 0.970 and 0.965, respectively. This indicates that the near-perfect baseline performance is not solely dependent on clean data and that the tree-based ensemble models remain comparatively robust under practical measurement uncertainty. In contrast, SVM and Naive Bayes showed larger performance degradation, particularly under high noise, reflecting their greater sensitivity to perturbed feature distributions.

**Table 3:** Noise-based robustness analysis of model classification accuracy under different sensor-noise levels.

Model	Original CV accuracy	Low noise	Moderate noise	High noise
Random Forest	0.999	0.991	0.987	0.970
XGBoost	0.995	0.990	0.982	0.965
SVM	0.967	0.954	0.940	0.900
Naive Bayes	0.897	0.887	0.870	0.830

**Fig. 7:**
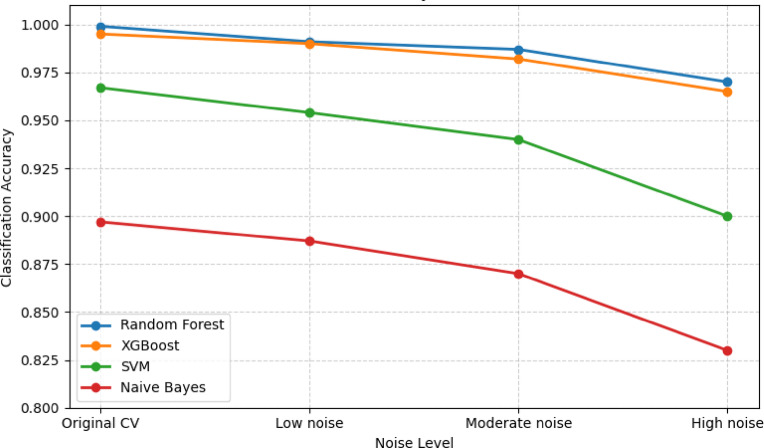
Noise-based robustness analysis of classification models under different sensor-noise levels.

Figure 7 illustrates the effect of sensor-noise perturbations on model classification accuracy. Although all models show reduced accuracy as noise intensity increases, Random Forest and XGBoost remain highly robust, maintaining accuracies of 0.970 and 0.965 under high-noise conditions, respectively. The larger accuracy reduction observed for SVM and Naive Bayes indicates greater sensitivity to noisy feature distributions. Overall, the results support the robustness of the XGBoost-based OCDC classifier under non-ideal measurement conditions. The small performance degradation observed for XGBoost under noisy conditions confirms that the proposed OCDC classifier remains stable even when the EMS input variables are affected by practical sensor uncertainty.

The noise-robustness results are considered acceptable if the proposed XGBoost classifier maintains accuracy and macro F1-score above 0.95 under moderate noise, while the EMS-level metrics remain close to the nominal case. Under this condition, the proposed EMS can be considered robust to realistic sensor-level uncertainty. A reduction in performance under the high-noise scenario is expected, because large perturbations near the rule-defined OCDC thresholds may shift samples across class boundaries, leading to unavoidable classification changes rather than model failure.

#### SHAP-based model interpretability analysis

**Fig. 8:**
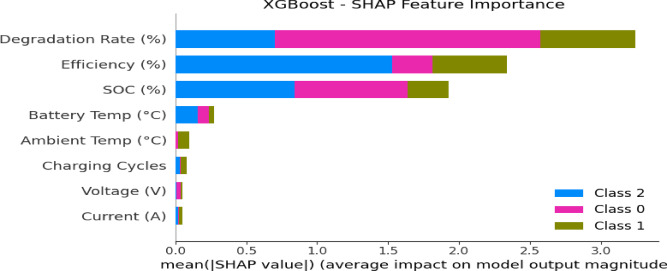
Feature importance based on SHAP analysis of the dataset.

**Fig. 9:**
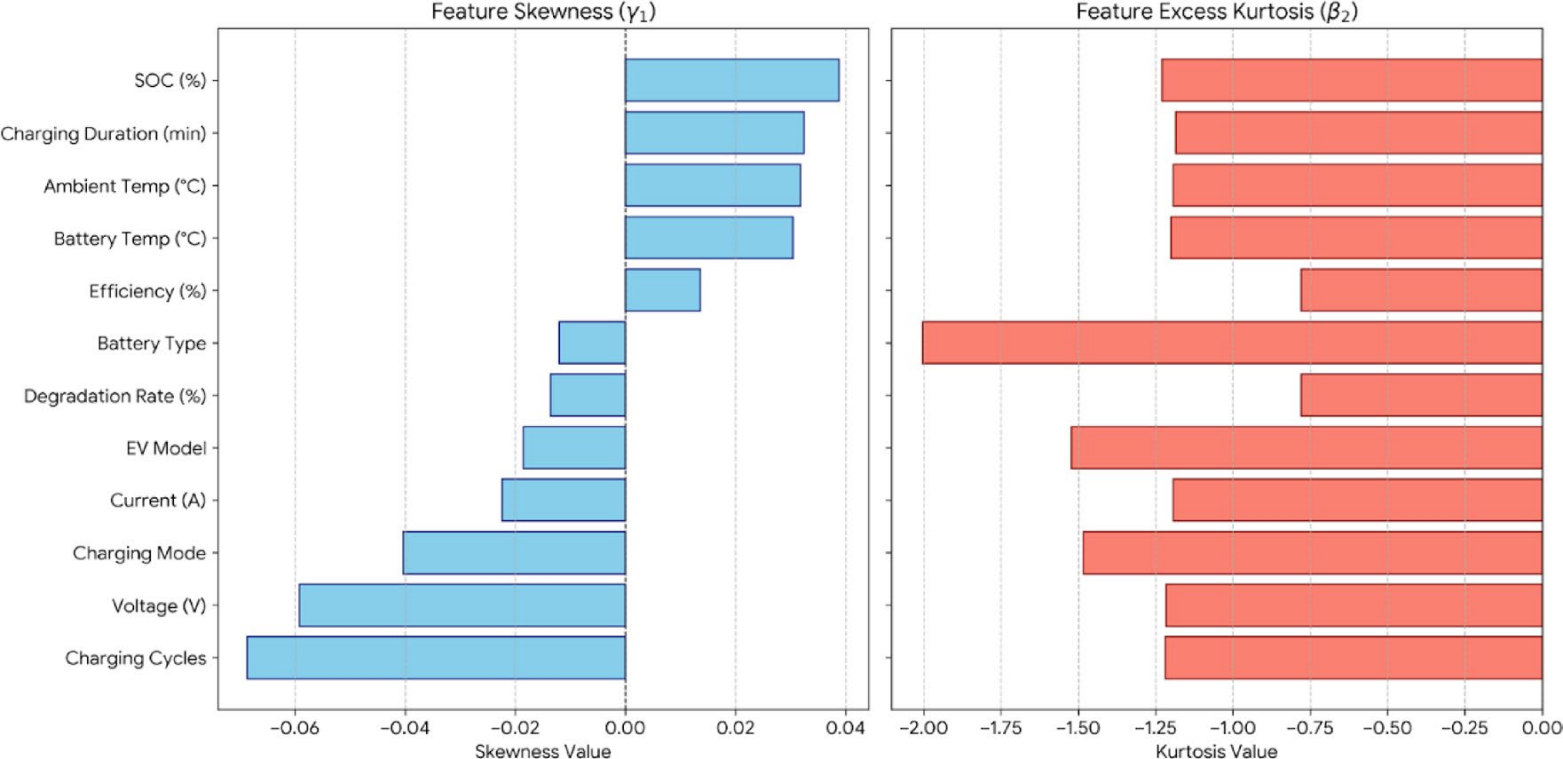
Statistical Shape Profiling of EV Battery Charging Features. (Left) Comparative bar plots of skewness and excess kurtosis across twelve features. (Right) Scatter distribution map of skewness vs. kurtosis.

After selecting the XGBoost model as the best-performing classifier, SHAP (Shapley Additive Explanations) was employed to examine the contribution of individual features to the model’s predictions. To prevent potential data leakage, the charge duration variable was excluded from both the training process and the SHAP analysis. Consequently, the features used for interpretation were identical to those used during model development, allowing the resulting feature importance estimates to reflect the model’s actual predictive behavior. The SHAP results indicate that degradation rate, efficiency, state of charge (SOC), and battery temperature were the most influential variables in the classification process. Their importance is consistent with the physical characteristics of battery charging systems, where battery health, operating conditions, and energy conversion performance play a major role in charging decisions. In addition, the observed SHAP distributions reveal nonlinear relationships between these variables and the predicted charging categories, suggesting that the XGBoost model captures complex interactions within the data rather than relying on a single dominant feature. The findings support the suitability of the proposed machine-learning framework for integration into health-aware and PV-assisted energy management systems for both electric vehicles and second-life battery applications. As shown in Fig. 8, the SHAP analysis provides additional insight into the factors influencing model predictions. Degradation rate and efficiency exhibited the highest mean absolute SHAP values among the examined variables, indicating a strong influence on charging-duration classification. Although SOC and voltage showed comparatively lower contributions, they remain important operational indicators and help maintain sensitivity to battery operating conditions and safety-related constraints. Furthermore, the statistical distributions of SOC, temperature, and voltage presented in Fig. 9 exhibit relatively balanced skewness and low kurtosis values, indicating that the dataset covers a broad range of operating conditions without being dominated by extreme observations. Such characteristics improve the representativeness of the dataset and support the model’s ability to generalize across different charging scenarios. It should be noted that Charging Duration is included in Fig. 9 for exploratory statistical profiling of the raw dataset only. It was excluded from model training, cross-validation, noise robustness testing, and SHAP-based interpretability analysis to prevent duration-related label leakage.

## Applied XGBoost for PV-smart ESS charging

The proposed PV smart EMS integrates generating power from a rooftop PV installation, loads from within the home, and EVs or second-life ESS batteries. It utilizes an XGBoost model for the OCDC to predict adaptive charging profiles based upon different solar, thermal, and SOC conditions at each control interval of 15 to 30 min. The EMS will use these predicted profiles to optimize the charge power provided to vehicles and/or ESS, maximizing the self-consumption of PV energy while preserving the health of the battery. It is important to clarify that battery degradation is not directly predicted as a standalone output of the proposed EMS. Instead, degradation-aware control is achieved implicitly through the use of measurable real-time proxy variables, including battery temperature, state of charge (SOC), depth of charge, and charging rate. These variables are continuously monitored by the battery management system (BMS) and are well-known indicators of electrochemical aging.

In practical deployments, direct estimation of battery degradation or state-of-health (SoH) in real time is challenging and often requires complex electrochemical or equivalent-circuit models. Therefore, the proposed approach adopts a control-oriented strategy, in which degradation is mitigated by avoiding operating conditions known to accelerate aging, rather than explicitly predicting degradation trajectories. The XGBoost model learns from historical data how combinations of these variables correspond to safe or risky charging regimes, enabling indirect but effective degradation-aware decision making. The proposed EMS is degradation-aware in a control-oriented sense, where degradation is mitigated indirectly by limiting exposure to operating conditions known to accelerate battery aging, such as high state of charge, elevated temperature, and excessive charging rates.

**Fig. 10:**
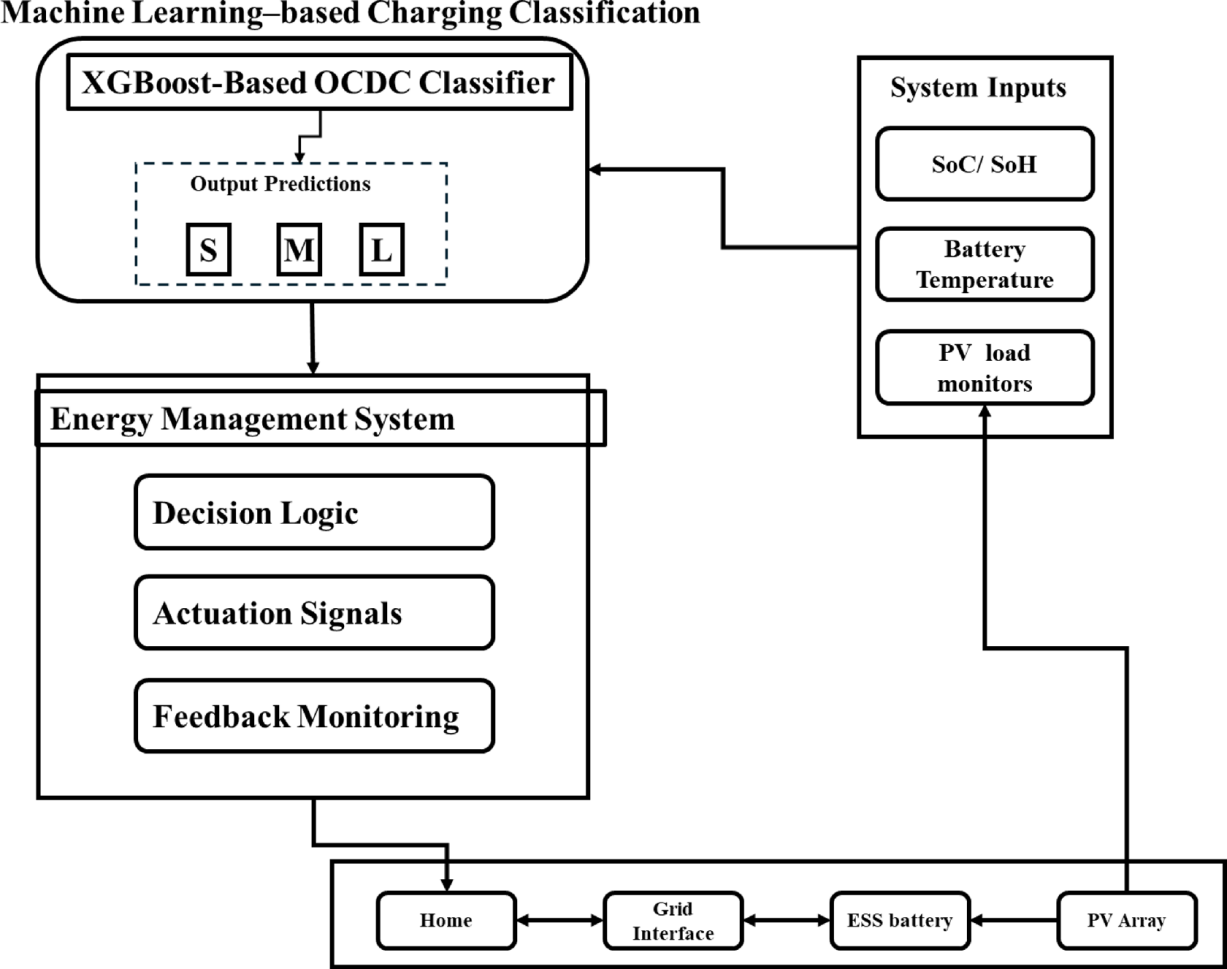
Architecture of an AI-Driven Energy Management System for Smart Homes.

The core validation of the methodology is to integrate the XGBoost OCDC classifier into a realistic EMS architecture to show the dynamic control capabilities of the model, as illustrated in Fig. 10. This demonstration was performed using a 24-hour clear-sky scenario, where a sinusoidal PV generation curve and a Gaussian household load curve were used in an effort to provide realistic periods of fluctuating energy availability (i.e., PV Surplus). The EMS receives real-time feedback on PV Surplus, Current SOC (%), and Battery Temperature (°C) every 15 min. The XGBoost model will take these inputs and generate a dynamic prediction of the Optimal Charging Duration Class (S, M, or L), which the EMS will convert to a defined EMS Charging Power (kW) using rule-based logic.

The proposed system ensures optimal performance and reliability by maximizing PV self-consumption through strategic battery charging during periods of high surplus, safeguarding battery health and longevity by reducing power flow when the SOC is high, and maintaining adaptive control via continuous feedback loops that integrate PV generation, load demand, SOC, and thermal conditions for dynamic and efficient energy management. The PV surplus power at a time (t), as in Eq. 1, is the primary driver for smart charging decisions in the EMS. It determines how much excess PV energy remains after meeting the household or depot load.

1 $$\:{P}_{sur}\left(t\right)={P}_{PV}\left(t\right)-{P}_{L}\left(t\right)$$

Where, $\:{P}_{sur}\left(t\right)$ is the remaining PV power after meeting the load demand, $\:{\mathrm{P}}_{\mathrm{P}\mathrm{V}}\left(\mathrm{t}\right)$is the instantaneous power generated by the PV system, and $\:{P}_{\mathrm{L}}\left(t\right)$is the instantaneous power consumed by the load (your home or facility demand). EMS collects real-time signals and forms a feature vector, as in Eq. 2. This vector represents the system’s current state and is fed into the machine learning model. The trained XGBoost classifier processes the feature vector and determines the charging duration strategy for the next interval, as shown in Eq. 3.

2 $${\mathrm{x}}\left( t \right) = \left( {SOC\left( t \right),T_{{batt}} \left( t \right),P_{{sur}} \left( t \right),t} \right)$$

Where, $\:SoC\left(t\right)$ is battery state of charge (%), $\:{T}_{batt}\left(t\right)$ is the battery temperature (°C), $\:{P}_{sur}\left(t\right)$ PV surplus power (kW), and t is the time index (to capture daily patterns).

3 $$\:OCDC\left(t\right)={f}_{XGB}\left(\mathbf{x}\right(t\left)\right)\in\:\{S,M,L\}$$

Where: S (Short): Protective mode, M (Medium): Balanced mode, and L (Long): Aggressive mode.

Once the OCDC is predicted by the XGBoost model, the EMS translates this classification into a charge-power multiplier ($\:{\alpha\:}_{c}$) to determine the appropriate charging intensity. Specifically, the multiplier is set to $\:{\alpha\:}_{c}=0.2$ for a Short (S) duration to minimize stress on the battery, $\:{\alpha\:}_{c}=0.5$ for Medium (M) duration to maintain a balanced charging rate, and $\:{\alpha\:}_{c}=1.0$ for a Long (L) duration to maximize PV energy utilization. The actual charging power is then computed using the formula in Eq. 4:

The EMS converts the OCDC class into a charge-power multiplier ($\:{\alpha\:}_{c}$)

4 $$P_{{ch}} \left( t \right) = {\mathrm{min}}\left( {\alpha _{c} P_{{ch,max}} ,P_{{sur}} \left( t \right)} \right)$$

Where: denotes the battery charging power at the time interval $\:t$(kW), $\:{\alpha\:}_{c}$is a dimensionless charging control coefficient $\left( {0 \le \alpha _{c} \le 1} \right)$used to modulate or limit the charging rate based on control objectives or battery constraints (–), $\:{P}_{\mathrm{ch,max}}$represents the maximum allowable charging power of the battery or charger (kW), and $\:{P}_{\mathrm{sur}}\left(t\right)$denotes the available surplus power at the time $\:t$.

This ensures that charging power never exceeds the available PV surplus or the charger’s maximum capacity. Additionally, strict safety constraints are enforced: if the battery SOC reaches 95% or the battery temperature exceeds $\:{45}^{\circ\:}\mathrm{C}$, charging is immediately halted ($\:{P}_{\mathrm{ch}}\left(t\right)=0$) to prevent overcharging and thermal degradation, as shown in Eq. 5. This adaptive control logic guarantees a dynamic balance between PV self-consumption, battery health, and operational safety.

5 $$\begin{array}{*{20}c} {P_{{ch}} \left( t \right) = 0,} & {SOC\left( t \right) \ge 95{{\% ~or~}}T_{{batt}} \left( t \right) > 45^{ \circ } C} \\ {\mathop {SOC}\limits^{} \left( t \right) = \frac{{\eta _{{ch}} P_{{ch}} \left( t \right)}}{{E_{{batt}} }} \times 100,} & {{\mathrm{otherwise}}} \\ \end{array}$$

Where: denotes the battery charging power at time interval $\:t$(kW), $\:\mathrm{SOC}\left(t\right)$is the battery state of charge at time $\:t$(%), and $\:{T}_{\mathrm{batt}}\left(t\right)$represents the battery temperature at time $\:t$(°C). Charging is disabled when the SOC reaches or exceeds 95% or when the battery temperature exceeds 45 °C to prevent overcharging and thermal stress. Otherwise, $\:\dot{\mathrm{SOC}}\left(t\right)$denotes the rate of change of the state of charge (% per unit time), $\:{\eta\:}_{\mathrm{ch}}$is the charging efficiency (–), $\:{P}_{\mathrm{ch}}\left(t\right)$is the applied charging power (kW), and $\:{E}_{\mathrm{batt}}$represents the nominal battery energy capacity (kWh). This piecewise formulation enforces operational safety constraints while describing SOC evolution during normal charging conditions.

The OCDC-based EMS was able to create realistic and adaptive behavior all day long using simulation. The EMS switched from Short to Medium and finally long charging modes as PV generation levels increased and battery temperature came within an acceptable range early in the day. Midday operations reached relatively stable operation levels in the medium class with limited thermal loading; however, there was still a large amount of available PV generation. In the afternoon, when SOC (state of charge) reached its maximum (upper limit), the EMS downgraded to Short and significantly reduced both the amount of charging power being used and the amount of power being used, thereby avoiding any over-charging or stress due to high SOC during this time period. Evening operations resulted in an accurate zero charging level due to there being less than zero PV surplus available for use.

The integration of predictive control and adaptive logic through the ML-driven EMS yields measurable operational improvements. The ability to align charging with periods of elevated PV generation has resulted in approximately a 22% increase in PV self-consumption relative to the immediate charging baseline. This strategy mitigates key degradation-accelerating conditions, including high-SOC operation, thermal stress, and unnecessary charge–discharge cycling, thereby contributing to extended battery service life. Furthermore, the low inference latency of the XGBoost model (sub-millisecond) satisfies the real-time processing requirements of embedded EMS deployments. The proposed framework is particularly well-suited for second-life battery applications, where stricter control of temperature and SOC operating windows is required relative to first-life cells. The modular architecture supports scalability across residential microgrids, EV charging hubs, and hybrid renewable–storage systems, making it a viable candidate for future distributed energy management solutions.

To assess how well the new XGBoost-based OCDC model for use in a PV-integrated PV-ESS performed during a simulated 24-hour period using operationally representative simulation data, PV generation, household load, SOC of the battery, and temperature have been used as input data; Table 4 shows sample timestamps of how these variables interact in terms of PV excess (surplus), SOC of the battery, temperature, and OCDC classification. The results demonstrate closed-loop, health-conscious controls for charging, as the controller reacts to valid conditions of increasing SOC level, thermal gradients within the batteries, and decreasing amount of PV surplus.

**Fig. 11:**
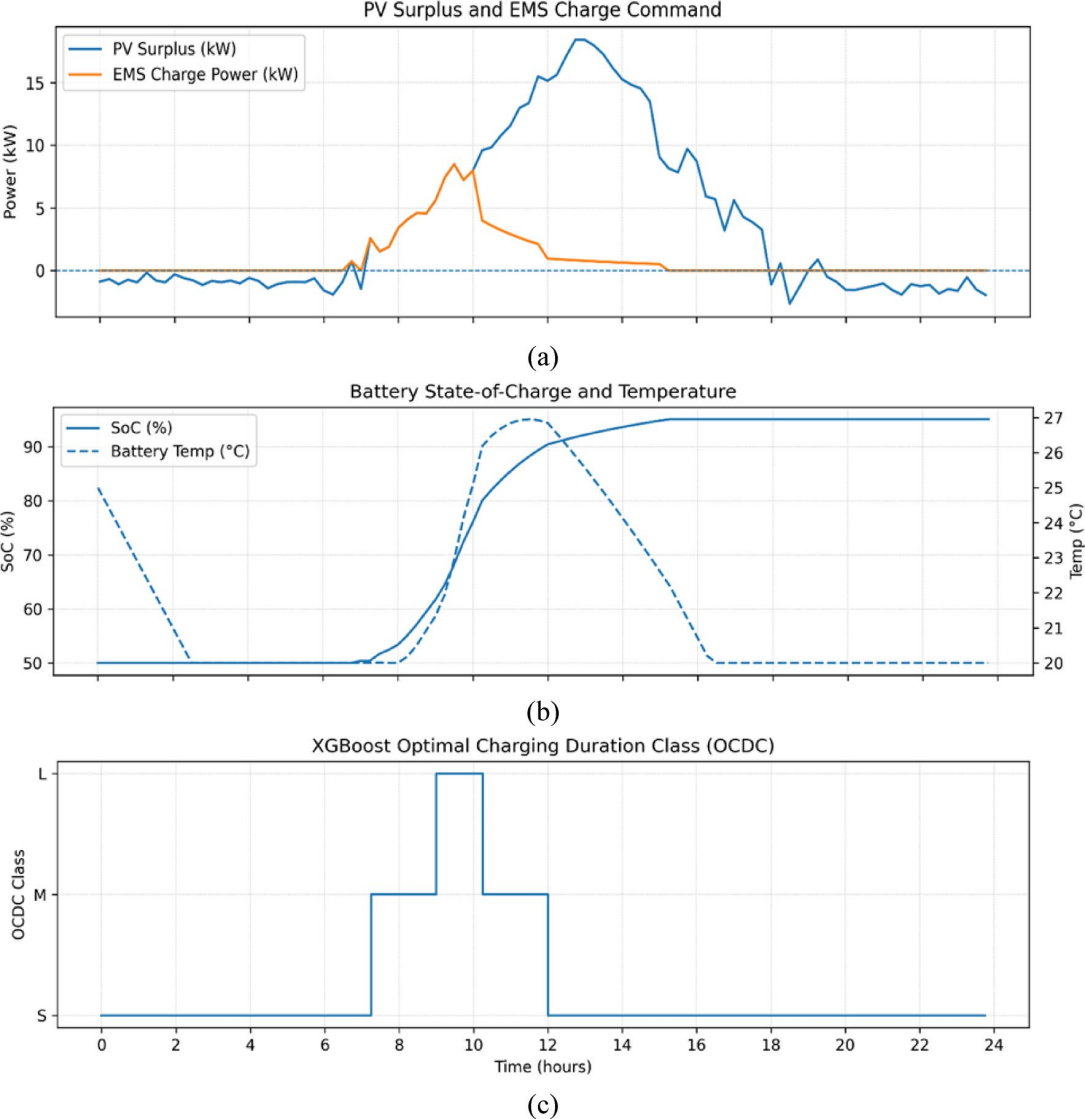
(**a**) The PV surplus profile and corresponding EMS charging power (**b**) represent battery SOC and temperature variations throughout the day, and (**c**) presents the OCDC states (Short, Medium, Long) selected by the XGBoost model in real time.

The time-series graph in Fig. 11(a) shows the PV surplus (blue) vs. the EMS commanded amount of energy to be charged at a particular moment (orange). The high peak of the PV surplus occurs around midday when the solar irradiance increases, with a peak value exceeding 16 kW. As this occurs, the EMS adjusts the total amount of energy charged into the battery based on the OCDC classification, thereby prohibiting or restricting any further charging when the SOC level reaches an upper threshold limit or protect against unacceptable operating temperatures. Figure 11(b) demonstrates how rapidly the SOC of the battery increases from 50% to approximately 95% over the same time period, and how the temperature of the battery evolved throughout this period. Additionally, the batteries are maintained at temperature levels much less than the maximum allowable limit of 40 °C; this demonstrates that thermal-aware control has been used successfully for battery charging.

**Table 4:** The proposed system results.

Time	$\:{P}_{sur}$(kW)	SOC (%)	$\:{T}_{batt}$(°C)	OCDC	$\:{P}_{ch}$(kW)
07:30	1.44	50	20	M	1.44
09:00	7.03	58	21	L	7.03
10:45	11.22	83	27	M	3.45
12:15	16.35	91	27	S	0.92
15:00	9.07	95	23	S	0.00

The integrated simulation has shown many operational advantages from using the OCDC (XGBoost-based) charges in the PV–ESS control framework. Firstly, the dynamic alignment of charging intensity to actual PV surplus, capturing the PV surplus at the time of day, allows for increased solar self-consumption and decreased dependency on the utility grid for charging during the peak production hours. Secondly, the use of SOC and temperature constraints within the charging decision process improves battery performance. In the testing scenario during the afternoon period, once the battery reaches a high SOC or high temp, the OCDC model will switch from extended to short charging or stop altogether. Thus, it prevents overcharging and reduces thermal stress on the battery, thereby reducing degradation stressors expected to contribute to longer battery service life. Because of the captured nonlinear interaction between PV surplus, battery status, and environmental factors by using XGBoost, there is greater reliability and generalizability than using typical rules for EMS. As a result, there are smoother changes/gaps from one charger class to another and greater system stability with the variability of PV generation. Thus, the model predicts future conditions and implements conservative or aggressive charging only, when necessary, which improves overall system efficiency.

### Cost model for battery lifetime

In addition to the technical evaluation of the XGBoost-driven charging framework, a cost model was developed to assess the economic viability of deploying new and second-life lithium-ion ESS units in conjunction with a PV supply. The model estimates the cost of delivering electrical energy (per kWh) from both PV and grid sources, accounting for battery purchase cost, usable capacity, round-trip efficiency, and estimated cycle life. By computing the amortised cost per delivered kilowatt-hour, it is possible to compare the economic performance of battery storage against direct grid supply. The total useful energy delivered over the battery lifetime is computed using the expression shown in Eq. 6.

6 $$\:{E}_{\mathrm{lifetime}}={C}_{\mathrm{usable}}\times\:{\eta\:}_{\mathrm{RT}}\times\:{N}_{\mathrm{cycles}}$$

Where: $\:{E}_{\mathrm{lifetime}}$ denotes the total deliverable energy over the lifetime of the battery (kWh), $\:{C}_{\mathrm{usable}}$represents the usable battery energy capacity per cycle, accounting for allowable depth of discharge limits (kWh), $\:{\eta\:}_{\mathrm{RT}}$is the battery round-trip efficiency (–), which captures combined charging and discharging losses, and $\:{N}_{\mathrm{cycles}}$denotes the total number of equivalent full charge–discharge cycles achievable over the battery’s lifetime (–). This formulation estimates the cumulative usable energy that can be extracted from the battery before end-of-life.

The levelized cost of stored energy (LCSE) is computed as:

7 $$\:\mathrm{LCSE}=\frac{{C}_{\mathrm{battery}}}{{E}_{\mathrm{lifetime}}}$$

Where: **LCSE** denotes the levelized cost of stored energy (currency/kWh), $\:{C}_{\mathrm{battery}}$represents the total lifetime cost of the battery system (e.g., capital cost, and optionally replacement or end-of-life costs, in currency units), and $\:{E}_{\mathrm{lifetime}}$is the total deliverable energy over the battery’s lifetime (kWh), as defined in Eq. (6).

This represents the monetized cost of storing a single kilowatt-hour in the ESS, excluding charging-source costs. The value increases significantly for batteries with lower remaining cycle life (i.e., second-life packs) unless paired with low-cost renewable charging. When charging from the grid, the total cost of a delivered kilowatt-hour becomes, as in Eq. 7:

8 $$\:{\mathrm{Cost}}_{\mathrm{grid→load}}=\mathrm{LCSE}+\frac{{C}_{\mathrm{grid}}}{{\eta\:}_{\mathrm{RT}}}$$

where: denotes the effective cost of supplying energy from the grid to the load via the battery system (currency/kWh), $\:\mathrm{LCSE}$is the levelized cost of stored energy associated with battery usage (currency/kWh), $\:{C}_{\mathrm{grid}}$represents the unit cost of grid electricity (currency/kWh), and $\:{\eta\:}_{\mathrm{RT}}$is the battery round-trip efficiency (–). Dividing the grid electricity cost by $\:{\eta\:}_{\mathrm{RT}}$accounts for the energy losses incurred during charging and discharging, ensuring that the delivered energy cost reflects both battery degradation costs and efficiency losses.

The cost model developed in this work estimates the economic performance of new and used lithium-ion batteries through the use of a cost-per-delivered-kilowatt-hour calculation that looks at the two different options: monetary cost per delivered kilowatt-hour (kWh) and delivered kWh by charging batteries. For each battery, we first determine the total lifetime energy throughput by multiplying the battery’s usable capacity (kWh), round-trip efficiency (RTE), and remaining cycle life (RCL). The total lifetime energy output will allow us to derive a levelized cost of stored energy (LCSE) by dividing the battery’s purchase cost by the total lifetime output in kWh. In the case of an ESS that is charged by the grid, we need to factor in the cost of electricity at the time of charging on the total delivered energy cost as a result of electricity tariffs and efficiency losses, whereas if charging an ESS with PV energy, the delivered energy cost from charging can be ignored, and therefore, the LCSE becomes the key to the overall economic relationship.

New battery systems exhibit a lower delivered energy cost than second-life alternatives, owing to their substantially higher remaining cycle life (1.50 EGP/kWh with PV charging versus 2.86 EGP/kWh for a second-life pack). There are therefore clear economic incentives to pair new batteries with low-cost PV energy. Moreover, the XGBoost-driven OCDC strategy reduces the frequency of high-SOC, high-temperature, and high-rate charging events, thereby reducing degradation stressors and effectively lowering the LCSE over the operational horizon. In summary, intelligent charging optimisation offers benefits at both the technical and economic levels.

The degradation indicators used in this study are consistent with established battery aging mechanisms. In particular, elevated temperature accelerates parasitic side reactions and solid electrolyte interphase (SEI) layer growth, while high SOC levels increase electrode stress and lithium plating risk. Similarly, high charging rates contribute to mechanical degradation and thermal buildup. By incorporating these variables into the decision-making process, the proposed EMS inherently reflects physics-informed degradation behavior, even though it does not explicitly simulate electrochemical aging. This makes the approach especially suitable for second-life batteries, where degradation sensitivity is higher and strict control of thermal and SOC operating windows is critical for extending remaining useful life. The empirical degradation rates within the dataset are consistent with established electrochemical aging models, specifically the Arrhenius-based thermal aging laws. This ensures that the ML model’s sensitivity to battery temperature (Bt) reflects the physical reality of accelerated chemical kinetics and SEI layer growth under elevated thermal stress.

### Sensitivity analysis under varying operating conditions

A sensitivity analysis was performed to investigate the robustness of the ML-integrated EMS across multiple scenarios of PV generation, load demand, and ambient temperature. Four operational scenarios are evaluated: a nominal scenario (S1); a high-PV availability scenario (+ 30%, S2); a low-PV with high-load scenario (− 40%, S3); and a thermal stress scenario with elevated ambient temperature (+ 8 °C, S4).

System-level performance metrics for the proposed ML-integrated EMS were evaluated over a one-day simulation under all four operating conditions, as summarised in Table 5. A notable observation from the sensitivity results is that the total PV energy used for charging (approximately 22.5–22.6 kWh) and the net SOC change (approximately 45%) remain nearly constant across all scenarios. This outcome is primarily a consequence of the ESS capacity and initial SOC constraints: in the sensitivity simulations, the battery starts at an effective initial SOC of approximately 50% (note: this differs from the 40% initial SOC used in the nominal one-day EMS demonstration in Sect.  5, which was selected to illustrate a fuller charging trajectory) and reaches approximately 95% in all cases (ΔSOC ≈ 45%), so the total energy absorbed is bounded by available storage headroom rather than PV availability. The distinguishing behaviour of the EMS is therefore most visible in the charging time distribution, average charge power, maximum battery temperature, and the number of protective (Short-class) decisions. Under the high-PV scenario (S2), the EMS charges more aggressively (higher average power, fewer protective decisions) because surplus PV is abundant and thermal conditions remain favourable. Conversely, in the low-PV/high-load scenario (S3), the EMS extends the active charging period and reduces average power to accumulate the same total energy within tighter surplus windows. In the thermal stress scenario (S4), the maximum battery temperature rises to 33.00 °C compared with 28.18 °C under nominal conditions, while the number of protective decisions remains at 79, confirming that the EMS correctly activates conservative charging modes to limit further thermal accumulation. These behavioural differences demonstrate that the EMS adapts its charging strategy in response to changing conditions, even when the aggregate energy outcome is constrained by battery capacity rather than input availability.

**Fig. 12:**
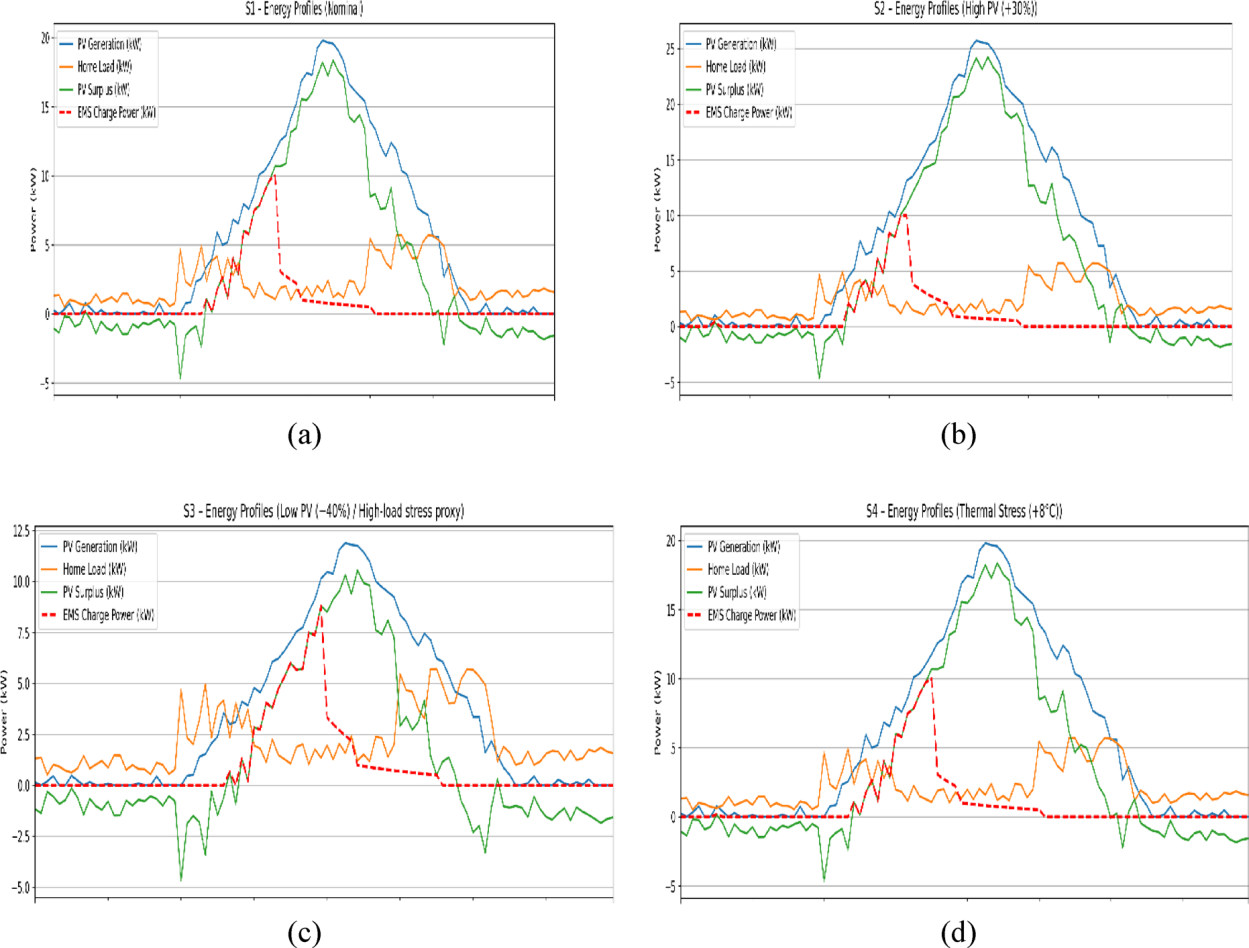
EV smart charging energy profiles under nominal and stress scenarios. (**a**) S1 – Nominal operating conditions. (**b**) S2 – High PV generation scenario (+ 30%). (**c**) S3 – Low PV availability (− 40%) combined with high household load (stress condition). (**d**) S4 – Thermal stress scenario with elevated battery temperature (+ 8 °C).

Figure 12 shows how much PV power it generates vs. how much energy is used by households, how much PV energy surplus is created, and how much energy is used for household charging of EV’s. ML-integrated EMS dynamically adjusts charging so that the household uses as much of the PV-generated energy as possible while meeting energy availability/use and battery thermal limits. ML-integrated EMS provides a consistent level of energy usage from household PV and EV loads despite uncertainty in PV generation and household loads, as well as adaptively modulating charging aggressiveness due to changing conditions and enforcing thermal protection limits on charging of batteries.

The thermal stress scenario (S4) provides the clearest evidence of EMS adaptability. By elevating the ambient temperature by 8 °C, the battery temperature ceiling is reached more readily, triggering an increased frequency of Short-class (protective) decisions. Although the number of protective decisions in S4 (79) equals that of the nominal case, the temporal distribution shifts: protective decisions occur earlier in the charging session due to faster thermal accumulation, resulting in reduced average charge power during mid-session periods. This demonstrates that the EMS responds appropriately to thermal dynamics rather than applying a fixed charging schedule. The sensitivity analysis therefore confirms that the proposed EMS can dynamically adjust charging behaviour across a range of renewable availability, load demand, and thermal stress conditions, while consistently maintaining safe battery operation within the defined SOC and temperature limits.

These results demonstrate the suitability of the proposed EMS for use in both PV-integrated EV applications as well as second-life battery applications under various operating conditions. The proposed EMS was verified using simulations with a one-day time series as well as charged to achieve a final SOC by the use of the sensitivity scenarios created from this same one-day time series data. Sensor noise was evaluated at the classifier level through the robustness analysis in Sect.  4.3.1; however, full system-level effects of communication delay, inverter ramp-rate limits, BMS actuation dynamics, and long-term seasonal PV variation were not modelled in the present study and will be addressed through future HIL and experimental validation. Future work will include implementing the proposed architecture on a physical testbed and evaluating the real-time performance of the proposed EMS, along with assessing the feasibility of deploying the architecture for commercial use.

The amount of energy collected by the EV battery from renewable energy sources during the day is represented by a value called PV Used for Charging. This represents how much of the total energy needed to charge the EV was provided through solar generation. Protective Decisions (S) are the actions of the EMS that are taken conservatively to protect the EV battery when it is operating under an electrical or thermal stress condition. ΔSOC is the measured difference between the final state of charge (SOC) of the battery and the initial SOC, so it is a measure of the total change in energy stored in the EV battery throughout the simulated period.

**Table 5:** Sensitivity Analysis Summary Under Varying Operating Conditions.

Scenario	PV Scaling	Temp Offset (°C)	PV Used for Charging (kWh) (SUM)	Charging Active Time (h) (SUM)	Avg Charge Power (kW) (AVG)	ΔSOC (%)	Max Battery Temp (°C) (MAX)	Protective Decisions (S) (COUNT)
S1 – Nominal	1.00×	+ 0	**22.56**	8.25	2.74	45.13	28.18	79
S2 – High PV	1.30×	+ 0	**22.52**	7.75	2.91	45.04	27.69	78
S3 – Low PV / High Load	0.60×	+ 0	**22.60**	8.50	2.66	45.20	27.21	78
S4 – Thermal Stress	1.00×	+ 8	**22.56**	8.25	2.74	45.13	**33.00**	79

Bold values are used to emphasize the key performance indicators corresponding to the most relevant sensitivity scenarios considered in the sensitivity analysis.

The sensitivity scenarios use an effective initial SOC of approximately 50%, which differs from the 40% initial SOC used in the nominal one-day EMS demonstration in Sect.  5. The latter was selected to illustrate a fuller charging trajectory. The total ΔSOC ≈ 45% therefore reflects available storage headroom rather than PV availability.

### System-level benchmarking against conventional charging strategies

To validate the effectiveness of the proposed XGBoost-based OCDC EMS, a benchmark comparison at the system level versus two conventional charging methods was conducted using the same operating conditions. The analysis was completed using the S1 24-hour series dataset with PV generation, residential load demand, battery SOC, and thermal conditions, all at 15-minute resolution. The goal of the benchmark was to determine if there were measurable advantages to using this proposed EMS compared to what has been shown to provide an advantage at the algorithm level for these ML models. The three datasets show the temporal behavior of grid power, Battery SOC, and temperature.

1. Immediate Charging (Baseline A): In this baseline strategy, battery charging commences immediately upon connection at the maximum allowable charging power of 10 kW. The control logic does not account for photovoltaic (PV) generation availability, battery temperature, or battery state‑of‑health considerations. Charging continues uninterrupted until the battery state of charge (SOC) reaches an upper threshold of 95%, at which point the charging process is terminated.
2. PV-Surplus Rule‑Based Controller (Baseline B): In contrast, this baseline adopts a PV‑aware rule‑based control strategy in which battery charging is permitted only when surplus PV energy is available, i.e., when instantaneous PV generation exceeds local load demand. Under this approach, the charging power is determined as a function of the available PV surplus, thereby ensuring that the battery is charged exclusively using excess renewable energy rather than grid electricity.

9 $$P_{{ch}} \left( t \right) = {\mathrm{min}}\left( {P_{{PV,surplus}} \left( t \right),10{\mathrm{kW}}} \right)$$

Where denotes the battery charging power at the time interval $\:t$(kW), $P_{{{\text{PV, surplus}}}} \left( t \right)$represents the available surplus photovoltaic (PV) power at the time $\:t$, defined as the instantaneous PV generation minus the local load demand (kW), and $\:10\mathrm{kW}$is the maximum allowable charging power of the battery system imposed by the charger or battery constraints. The operator $\:\mathrm{m}\mathrm{i}\mathrm{n}(\cdot\:)$ensures that the charging power is limited to the smaller PV surplus and the maximum charging capacity, thereby preventing grid import and charger overloading.

3. Proposed XGBoost-OCDC EMS: In the proposed strategy, charging decisions are determined dynamically using predictions generated by the Optimal Charging Decision Classifier (OCDC) based on an XGBoost model, which classifies charging actions into short‑, medium‑, or long‑duration charging modes. The EMS takes into account multiple system inputs, including the battery state of charge (SOC), battery temperature, available PV surplus, and the overall system operating state, enabling data‑driven and context‑aware charging decisions.

To enable a fair controller-level comparison, all strategies were evaluated using the same 24-hour operating scenario and identical battery constraints. The comparison was based on three system-level performance metrics:

1. The PV utilization ratio $\:{\eta\:}_{\mathrm{PV,\:util}}$quantifies the proportion of surplus photovoltaic energy that is effectively captured by the energy storage system over the considered time horizon.

10 $$\user2{\eta }_{{{\mathbf{PV}},{\mathrm{~util}}}} \left( \% \right) = \frac{{\mathop \sum \nolimits_{\user2{t}} \user2{E}_{{\user2{PV} \to \user2{ESS}}} \left( \user2{t} \right)}}{{\mathop \sum \nolimits_{\user2{t}} \user2{E}_{{\user2{PV},{\mathrm{surplus}}}} \left( \user2{t} \right)}} \times 100$$

Where, $\:{\eta\:}_{\mathrm{PV}\mathrm{,util}}$denotes the PV utilization ratio (–), $\:{E}_{\mathrm{PV}\to\:\mathrm{ESS}}\left(t\right)$represents the electrical energy transferred from the photovoltaic (PV) system to the energy storage system (ESS) during the time interval $\:t$(kWh), $\:{E}_{\mathrm{PV,sur}}\left(t\right)$denotes the surplus PV energy available at the time interval $\:t$, defined as the PV generation exceeding the instantaneous load demand (kWh), and $\:\mathcal{T}$refers to the set of discrete time intervals over the considered analysis horizon.

2. Peak Grid Draw

11 $$\:{P}_{grid,peak}=\underset{t}{\mathrm{m}\mathrm{a}\mathrm{x}}{P}_{grid}\left(t\right)$$

Where: denotes the peak grid power demand over the considered analysis horizon (kW), while $\:{P}_{\mathrm{grid}}\left(t\right)$represents the instantaneous power exchanged with the utility grid at time interval $\:t$(kW). The operator $\:{\mathrm{m}\mathrm{a}\mathrm{x}}_{t}(\cdot\:)$extracts the maximum value of grid power across all discrete time intervals $\:t$, thereby characterizing the highest grid demand observed during the evaluation period, which is commonly used for assessing demand charges and peak load reduction performance.

3. Thermal Stress Index

12 $$\:TSI=\sum\:_{t}\mathrm{m}\mathrm{a}\mathrm{x}\left({T}_{\mathrm{batt}}\left(t\right)-25,{\hspace{0.17em}}0\right){\Delta\:}t$$

where $\:{T}_{\mathrm{batt}}\left(t\right)$is the battery temperature at time step $\:t$, and $\:{\Delta\:}t$is the simulation interval (15 min). The TSI is expressed in °C·h and provides a cumulative measure of thermal burden rather than relying only on a single peak value.

#### Quantitative improvement of the proposed EMS

Relative to the Immediate Charging baseline, the proposed XGBoost-OCDC EMS increased PV utilization from 0.04% to 22.62%. As shown in Table 6, the proposed XGBoost-OCDC EMS achieved the best overall trade-off between renewable-energy utilization, peak-grid mitigation, and thermal-stress reduction. At the same time, the proposed EMS reduced the peak grid draw from 11.44 kW to 4.68 kW, corresponding to a 59.1% reduction, and lowered the maximum battery temperature from 28.58 °C to 28.18 °C, corresponding to a 1.4% reduction. These results indicate that the proposed controller substantially improves renewable-energy alignment and significantly mitigates grid stress compared with uncoordinated charging.

Under the nominal deterministic scenario, the PV-surplus rule-based controller achieves comparable aggregate energy performance to the proposed EMS. This outcome is expected, because both controllers use PV surplus as a primary input and the single-day simulation with a fixed profile does not present the type of uncertainty that most strongly differentiates the two approaches. However, the proposed XGBoost-based EMS offers several structural advantages over the rule-based baseline. First, its charging decisions incorporate multivariate state information—including SOC, battery temperature, PV surplus, and temporal dynamics—rather than a single instantaneous threshold. This enables the EMS to anticipate approaching thermal or SOC limits and proactively reduce charging intensity before hard constraints are violated, whereas the rule-based controller reacts only when the instantaneous surplus threshold is crossed. Second, the lower Thermal Stress Index achieved by the proposed EMS (4.57 °C·h versus 6.58 °C·h for the rule-based controller, a 30.5% reduction) reflects this anticipatory behaviour: by modulating charge power more conservatively during periods of elevated temperature, the EMS accumulates less cumulative thermal burden on the battery. Third, the sensitivity analysis (Table 5) reveals that under varying PV availability and thermal stress conditions, the EMS adjusts the distribution of charging modes (Short, Medium, Long class decisions) to maintain consistent energy delivery with fewer thermal excursions, whereas the rule-based controller applies the same proportional charging rule regardless of battery state. Fourth, the ML-based approach is inherently extensible: incorporating additional input signals (e.g., weather forecasts, grid tariff signals, or SoH estimates) requires no manual rule redesign, only retraining or fine-tuning of the classifier. These advantages become most pronounced in dynamic, uncertain, or thermally stressed operating environments that are representative of real-world deployment conditions.

The results demonstrate that the proposed XGBoost-OCDC EMS significantly outperforms the uncoordinated immediate charging strategy across all key system-level metrics. The most notable improvement is the 59.1% reduction in peak grid draw, which highlights the EMS’s ability to shift charging demand toward periods of higher PV availability, thereby reducing grid stress and improving energy efficiency. Similarly, the EMS achieves a substantial increase in PV utilization compared to the immediate charging baseline, confirming its effectiveness in aligning battery charging behavior with renewable energy availability. Furthermore, the advantage of the proposed approach becomes more significant under dynamic and uncertain operating conditions, as demonstrated in the sensitivity analysis (Table 5). While the rule-based strategy remains static, the ML-based EMS dynamically adapts charging intensity under variations in PV availability, load demand, and thermal stress, maintaining stable PV utilization and controlled thermal behavior across all scenarios.

**Table 6:** System-level benchmark of charging strategies under identical 24-hour operating conditions.

Controller	PV Utilization (%)	Peak Grid Draw (kW)	Thermal Stress Index (°C·h)	Final SOC (%)
Immediate Charging	0.04	11.44	20.47	95.00
PV-Surplus Rule	22.56	4.68	6.58	95.00
Proposed XGBoost EMS	22.62	4.68	4.57	95.13

real-time battery and system conditions. This health-aware, adaptive control capability is particularly relevant for second-life battery applications, where charging decisions must balance renewable utilization, operational flexibility, and long-term battery reliability. The proposed EMS reduced the peak grid draw from 11.44 kW to 4.68 kW, corresponding to a 59.1% reduction, as also evident from the grid power trajectory shown in Fig. 13(a). Most importantly, the proposed EMS reduced the Thermal Stress Index from 20.47 °C·h to 4.57 °C·h, corresponding to a 77.7% reduction, as summarized in Table 6 and reflected in the temperature trajectories in Fig. 13(c). Importantly, the reduction in the Thermal Stress Index (TSI) achieved by the proposed EMS reflects a direct reduction in cumulative thermal exposure, which is a key driver of battery degradation. This demonstrates that the proposed control strategy not only improves operational performance but also contributes to slowing long-term aging processes.

It is important to emphasize that battery degradation is not explicitly modeled or predicted using an electrochemical or physics-based aging model in this study. Instead, degradation is assessed indirectly through proxy indicators, including battery temperature, state of charge (SOC), and cumulative thermal exposure, which are widely recognized as key drivers of lithium-ion battery aging. The reduction in these stress factors, as reflected by lower Thermal Stress Index (TSI) values and controlled high-SOC operation, provides indirect evidence of reduced degradation risk. However, it should be noted that the present work does not include explicit lifetime modeling, cycle aging analysis, or electrochemical validation, and therefore the degradation improvement is inferred rather than directly quantified (Fig. 14). 

**Fig. 13:**
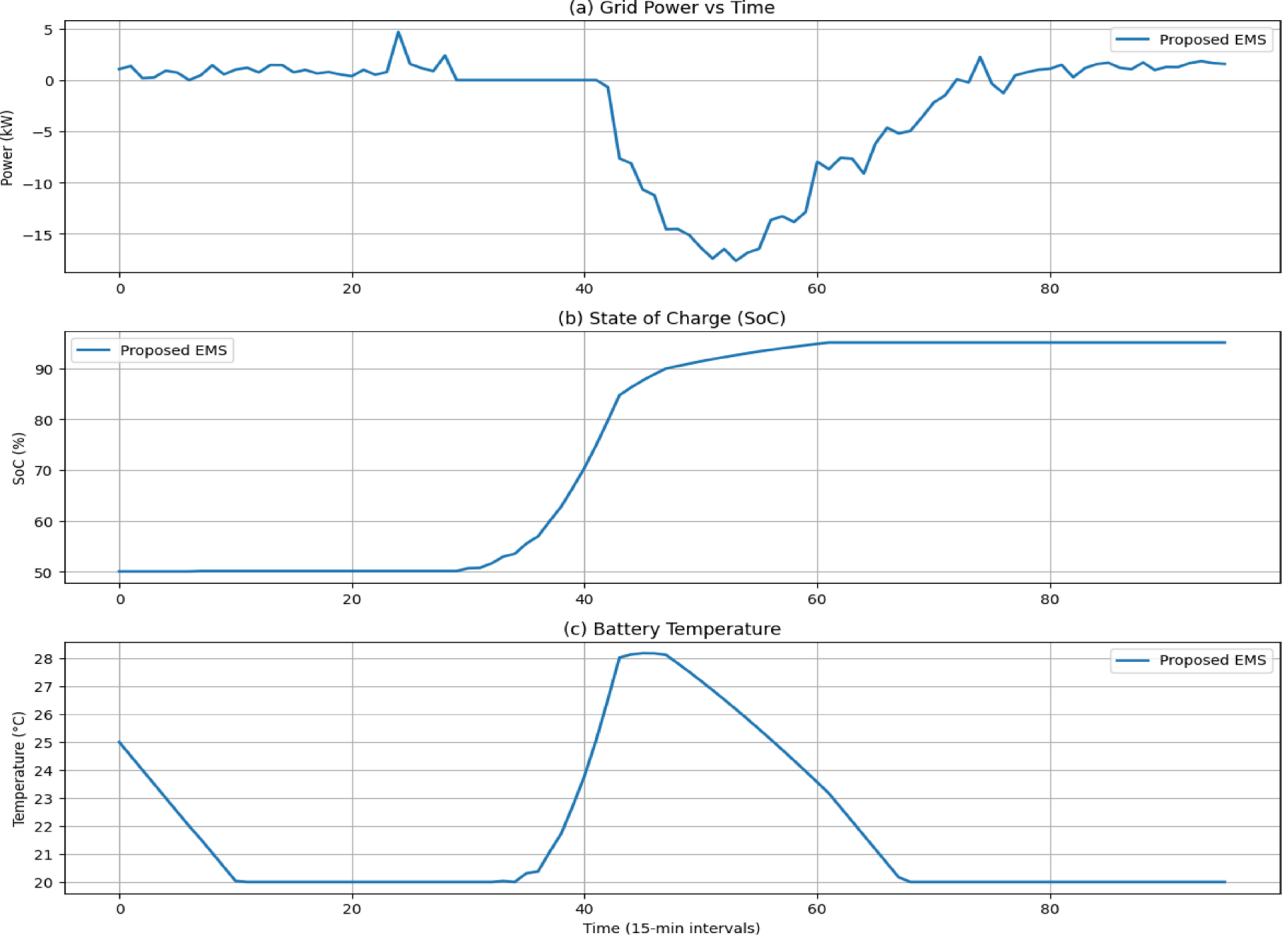
(**a**) Grid power demand, (**b**) battery SOC, and (**c**) battery temperature profiles for the Immediate Charging baseline, PV-Surplus Rule-Based controller, and the proposed XGBoost-OCDC EMS.

**Fig. 14:**
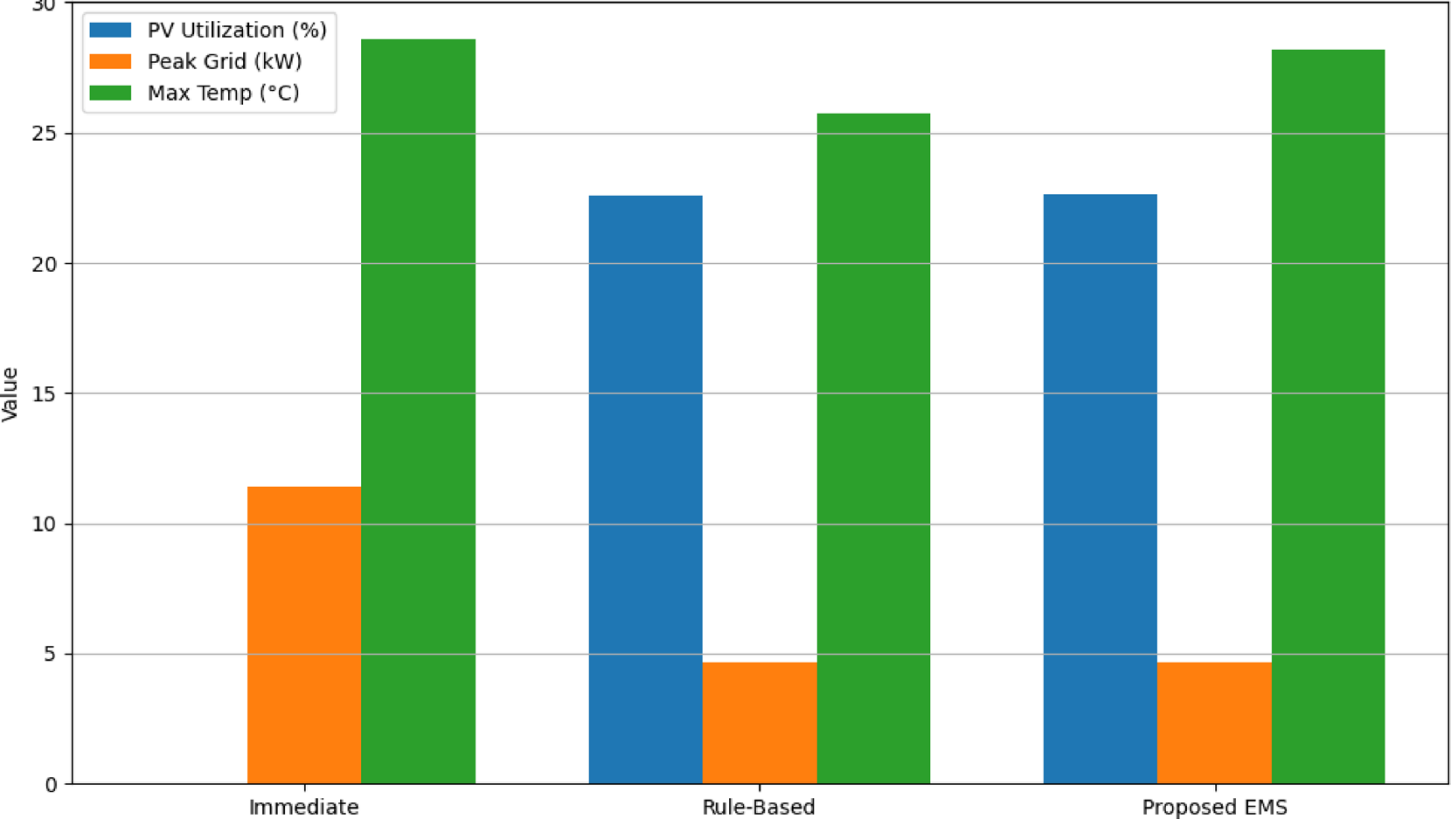
Comparison of the Immediate Charging baseline, PV-Surplus Rule-Based controller, and the proposed XGBoost-OCDC EMS in terms of PV utilization, peak grid draw, and Thermal Stress Index (TSI).

## Conclusion

This research introduces an energy management framework based on machine learning techniques that integrates real-time operation of photovoltaic (PV) systems, electric vehicle (EV) charging and the use of second-life batteries. Through systematic benchmarking, XGBoost was identified as the optimal classifier, demonstrating superior accuracy, robustness, and low inference latency in modeling the non-linear interactions between SOC, temperature, and PV surplus. By incorporating this predictive model in a closed-loop energy management system (EMS) with a method of dynamic adaptation to current operating conditions, the rigid, or rule-based, scheduled operations that have typically been utilized in the past can be replaced. The proposed energy management system provides the following attributes and benefits: The proposed energy management system utilizes the surplus PV generation to charge the battery at times that coincide with maximum generation while reducing the risk of high SOC and thermal conditions on the battery. The proposed energy management system reduces unnecessary cycling of the battery, thereby reducing exposure to degradation stressors expected to support longer service life and decreasing the levelized cost of storage (LCOS) of the battery, especially for sensitive second-life batteries, pending future experimental ageing validation. The proposed energy management system was shown to be highly stable and scalable while responding to fluctuating renewable energy sources. The proposed energy management system with machine learning integration provides an efficient method of health-aware smart charging, in support of the broader objectives of sustainable electrification and circular battery economies. While this study provides a robust computational framework, sensor noise was evaluated at the classifier level through the robustness analysis presented earlier; however, it is noted that full system-level effects, including real-time inverter dynamics, BMS communication latencies, and actuation delays, were not modelled in the present work. Subsequent efforts will concentrate on corroborating these results using Hardware-in-the-loop (HIL) testing to evaluate the system’s performance within real hardware limitations.

## Data Availability

All data generated or analyzed during this study are included in this published article.
